# Uncoupling the Excitatory Amino Acid Transporter 2 From Its C-Terminal Interactome Restores Synaptic Glutamate Clearance at Corticostriatal Synapses and Alleviates Mutant Huntingtin-Induced Hypokinesia

**DOI:** 10.3389/fncel.2021.792652

**Published:** 2022-01-31

**Authors:** Stefan Hirschberg, Anton Dvorzhak, Seyed M. A. Rasooli-Nejad, Svilen Angelov, Marieluise Kirchner, Philipp Mertins, Gilla Lättig-Tünnemann, Christoph Harms, Dietmar Schmitz, Rosemarie Grantyn

**Affiliations:** ^1^Synaptic Dysfunction Lab, Neuroscience Research Center, Charité – Universitätsmedizin Berlin, Corporate Member of Freie Universität Berlin, Humboldt-Universität zu Berlin, Berlin Institute of Health, Berlin, Germany; ^2^Proteomics Platform, Max Delbrück Center for Molecular Medicine in the Helmholtz Association, Berlin, Germany; ^3^Berlin Institute of Health (BIH), Berlin, Germany; ^4^Department of Experimental Neurology, Charité – Universitätsmedizin Berlin, Berlin, Germany; ^5^Center for Stroke Research Berlin, Charité – Universitätsmedizin Berlin, Berlin, Germany; ^6^German Center for Neurodegenerative Diseases (DZNE), Berlin, Germany; ^7^Cluster of Excellence NeuroCure, Berlin, Germany; ^8^Einstein Center for Neurosciences Berlin, Berlin, Germany

**Keywords:** astrocyte activation, Huntington’s disease (HD), EAAT2 interaction proteomics, synaptic glutamate clearance, corticostriatal pathology, hypokinesia

## Abstract

Rapid removal of glutamate from the sites of glutamate release is an essential step in excitatory synaptic transmission. However, despite many years of research, the molecular mechanisms underlying the intracellular regulation of glutamate transport at tripartite synapses have not been fully uncovered. This limits the options for pharmacological treatment of glutamate-related motor disorders, including Huntington’s disease (HD). We therefore investigated the possible binding partners of transgenic EAAT2 and their alterations under the influence of mutant huntingtin (mHTT). Mass spectrometry analysis after pull-down of striatal YFP-EAAT2 from wild-type (WT) mice and heterozygote (HET) Q175 mHTT-knock-in mice identified a total of 148 significant (FDR < 0.05) binders to full-length EAAT2. Of them 58 proteins exhibited mHTT-related differences. Most important, in 26 of the 58 mHTT-sensitive cases, protein abundance changed back toward WT levels when the mice expressed a C-terminal-truncated instead of full-length variant of EAAT2. These findings motivated new attempts to clarify the role of astrocytic EAAT2 regulation in cortico-basal movement control. Striatal astrocytes of Q175 HET mice were targeted by a PHP.B vector encoding EAAT2 with different degree of C-terminal modification, i.e., EAAT2-S506X (truncation at S506), EAAT2-4KR (4 lysine to arginine substitutions) or EAAT2 (full-length). The results were compared to HET and WT injected with a tag-only vector (CTRL). It was found that the presence of a C-terminal-modified EAAT2 transgene (i) increased the level of native EAAT2 protein in striatal lysates and perisynaptic astrocyte processes, (ii) enhanced the glutamate uptake of transduced astrocytes, (iii) stimulated glutamate clearance at individual corticostriatal synapses, (iv) increased the glutamate uptake of striatal astrocytes and (iv) alleviated the mHTT-related hypokinesia (open field indicators of movement initiation). In contrast, over-expression of full-length EAAT2 neither facilitated glutamate uptake nor locomotion. Together, our results support the new hypothesis that preventing abnormal protein-protein interactions at the C-terminal of EAAT2 could eliminate the mHTT-related deficits in corticostriatal synaptic glutamate clearance and movement initiation.

## Introduction

In the adult rodent striatum most of the glutamate transport is carried out by EAAT2 (gene name *Slc1a2*) (Danbolt, 2001; Beart and O’Shea, 2007; Vandenberg and Ryan, 2013). Clustering of the EAAT2 protein at the sites of synaptic glutamate release ensures the rapid return of the extracellular glutamate concentration to very low resting levels (Bergles and Jahr, 1998). In comparison with other synaptic proteins, including the postsynaptic glutamate receptors, EAAT2 is present in larger amounts (Danbolt, 2001). It has been a matter of much debate whether or not synaptic glutamate uptake can ever become insufficient in the healthy brain. Under pathological conditions, a relative deficiency of glutamate uptake could result from the following distinct mechanisms: (i) insufficient *Slc1a2* transcription, (ii) impaired membrane targeting of EAAT2, (iii) exaggerated intracellular degradation of EAAT2 and/or iv) altered transporter-substrate interactions. Heterologous expression studies with recombinant chimeric EAAT2 variants illuminated the possible significance of particular EAAT2 domains. It turned out that a region close to the distal end of the last transmembrane domain is relevant for the interaction with the transport substrate (Leinenweber et al., 2011), but it also affects the balance between cytoplasmic retention and membrane insertion (Kalandadze et al., 2002; Underhill et al., 2015). Due to detailed studies from the Zafra lab, it is known that ubiquitination of 4 C-terminal lysine sites (Gonzalez-Gonzalez et al., 2008) substantially contributes to the internalization of the transporter from the plasma membrane. Finally, it was reported that sumoylated toxic C-terminal fragments may inhibit the *Slc1a2* transcription in the astrocyte nucleus (Gibb et al., 2007; Foran et al., 2011; Rosenblum et al., 2017). All these studies imply that EAAT2 binding partners at the C-terminal end can participate in the regulation of glutamate uptake, but detailed information on the spectrum of EAAT2 interactors in health and disease is not yet available. This limits further understanding of EAAT2-related disease mechanisms.

Impairment of glutamate homeostasis due to insufficient EAAT2 expression or function has already been implicated in neurodegenerative disease. This also includes HD – an autosomal dominant neurodegenerative disease of monogenic origin. A trinucleotide (CAG) expansion of exon 1 in the mutant huntingtin gene (*mHTT*) on chromosome 4 causes abnormally long polyglutamine (polyQ) stretches in the already large huntingtin protein. *mHTT* is expressed not only in neurons but also in astrocytes (Faideau et al., 2010) where the loss of normal huntingtin function and additional effects of misfolded polyQ fragments can produce a variety of alterations (Mrzljak and Munoz-Sanjuan, 2015; Tabrizi et al., 2019; Zeitler et al., 2019), including the modification of other proteins (Wanker et al., 2019). The clinical picture of HD is characterized by progressive motor, cognitive and emotional disturbances (Tabrizi et al., 2013). A motor symptom of advanced HD in humans and most rodents with *mHTT* expression is hypo-/bradykinesia (Berardelli et al., 1999; Hart et al., 2013; Horton et al., 2019), whereas jerky uncontrolled spontaneous movements (chorea) are mostly observed at earlier stages of HD (Rosenblatt et al., 2003; Tabrizi et al., 2013). Thorough quantification of the hypo- vs. hyperkinetic aspects of motor performance is important for the pharmacological management of the disease (Hart et al., 2013). In general, phenotype progression is less faithfully predicted by the scores of chorea as compared to those of hypo-/bradykinesia (Rosenblatt et al., 2003; Tabrizi et al., 2013).

The origin of mHTT-related hypokinesia is not well understood. According to the information available from extracellular recordings or imaging of neuronal activity in a mouse model of HD, motor symptoms may evolve as a consequence of disinhibition and abnormal synchronicity in the corticostriatal pathway (Miller et al., 2012; Burgold et al., 2019). However, most studies point to functional uncoupling rather than enhanced glutamatergic input to the striatum (Plotkin and Surmeier, 2015; Reiner and Deng, 2018; Veldman and Yang, 2018). On the presynaptic side, there is a tendency for down-regulation of vGlut1 immunofluorescence (Rothe et al., 2015), decrease in vGluT1+ terminal numbers (Deng et al., 2013) and impairment of synaptic glutamate release at individual corticostriatal terminals (Dvorzhak et al., 2019). On the postsynaptic side, corticostriatal synaptic transmission might be affected by insufficient supply with brain-derived neurotrophic factor (Plotkin et al., 2014) or reduced signal transfer from distal dendrites (Carrillo-Reid et al., 2019). The duration of the NMDAR component of corticostriatal EPSCs could be prolonged (Dvorzhak et al., 2019), which might be due to a reduced glutamate uptake capacity of striatal astrocytes (Tong et al., 2014; Dvorzhak et al., 2016). Indeed, targeted expression of *mHTT* in astrocytes consistently impeded their glutamate uptake function (Shin et al., 2005; Bradford et al., 2009; Faideau et al., 2010; Meunier et al., 2016) and induced or exacerbated the motor symptoms of HD (Bradford et al., 2009; Meunier et al., 2016).

The present study pursued 2 major aims: (1) to identify the protein binding partners of full-length and C-terminal-modified EAAT2 in normal and mHTT-expressing mice and (2) to explore the functional consequences of modified EAAT2 expression. The underlying hypothesis would be that, in the abnormal environment of a polyQ astrocyte, EAAT2 can establish pathological protein-protein interactions. Bypassing the latter by removal of selected EAAT2 binding sites may increase the availability of glutamate transporter, restore the compromised glutamate clearance function of the corticostriatal pathway and improve locomotion.

## Materials and Methods

### Adeno-Associated Virus Plasmid Design and Vector Production

Supplementary Table 1 presents an overview of the used vectors, the sites of injections and the purpose of a chosen indicator. Plasmid pRcCMV-mYFP-EAAT2 and pRcCMV-mYFP-EAAT2-S506X were gifts from Christoph Fahlke and Arnd Baumann (Forschungszentrum Jülich). pRcCMV-mYFP-EAAT2 contains the expression cassette for the human *Slc1a2* fused to N-terminal mYFP. In pRcCMV-mYFP-EAAT2-S506X a serine at position 506 is point-mutated to generate a stop codon that causes the truncation of the last 68 amino acids of the C-terminus. This truncation does not interfere with the membrane insertion of the protein or glutamate transport (Leinenweber et al., 2011). In contrast, the mutation of four C-terminal lysine (Lys, K) residues to arginine (Arg, R) in the full-length EAAT2 has been shown to be critical for transporter internalization (Gonzalez-Gonzalez et al., 2008). All EAAT2 plasmids were derived from human EAAT2, with the understanding that there is a close similarity between the human and mouse *Slc1a2* homologs (Kirschner et al., 1994).

Four consecutive cycles of site-directed mutagenesis were applied to generate EAAT2-4KR. In brief, pRcCMV-mYFP-EAAT2 was amplified by PCR using the mutagenesis primers listed in Supplementary Table 1, and the template was subsequently destroyed by DpnI digest. For high-efficiency transformation, NEB C2987H alpha-competent *Escherichia coli* were incubated with the DpnI-digested PCR mix. Successful mutants were screened by Sanger sequencing (Eurofins Genomics, Köln). The expression cassettes for mYFP-EAAT2, mYFP-EAAT2-4KR, and mYFP-EAAT2-S506X were subcloned by restriction digest with BamHI and EcoRI and ligation into a preexisting adeno-associated virus (AAV) backbone [pAAV-gfaABC1D-ChR2(LCTC)-p2a-FP635-WPRE] to produce pAAV-gfaABC1D-mYFP-EAAT2-WPRE, pAAV-gfaABC1D -mYFP-EAAT2-4KR-WPRE, and pAAV-gfaABC1D -mYFP-EAAT2-S506X-WPRE. Monomeric mRuby was subcloned from pKanCMV-mRuby-10aa-H2B into the three AAV vectors with AgeI and BsrGI to facilitate detection of the transduced astrocytes and the EGFP-based iGlu*_*u*_* in the corticostriatal terminals of the same preparation. Control vectors were constructed by amplifying mRuby or mYFP by PCR adding a 3′ stop codon and a SalI restriction site and were then cloned into an AAV backbone to generate pAAV-gfaABC1D-mYFP-WPRE or pAAV-gfaABC1D-mRuby-WPRE. The plasmids used or modified for further use are listed in Supplementary Table 1. The AAV vectors were produced at the Vector Core Facility of the Charité - University Medicine or at UPenn Vector Core. We acknowledge the contribution of Viviana Gradinaru and Benjamin Devermann for generating the PHP.eB adeno-associated virus (AAV) serotype (Chan et al., 2017).

### Antibodies

The primary and secondary antibodies used for the quantification of WB or IR are also listed in Supplementary Table 1. For concentrations see the “Materials and Methods” sections “Quantitative Immunofluorescence (IF)” and “Western Blot Analysis of Striatal Lysates.” The EAAT2-Ab (Abcam, Cambridge, United Kingdom) was raised against a synthetic peptide within rat EAAT2 aa 550 to the C-terminus (C terminal) conjugated to keyhole limpet haemocyanin. The exact sequence is proprietary. Apart from several other C-terminus-directed Abs, we also tried Ab77039 and Ab203130 from Abcam were peptide fragments between aa143 and 239 were used as antigen but did not achieve the quality of immunostaining seen with Ab41621.

### Animals

All data was obtained from aged (14–18 mo) Z-Q175-KI mice, a widely used mouse model of HD (Menalled et al., 2012). The mice were obtained from CHDI (“Cure Huntington’s Disease Initiative,” see stock # 027410 of the Jackson Laboratory, Bar Harbor, United States). The applicable international, national and institutional guidelines for the care and use of the animals were followed. Study design, performance of experiments and statistical evaluation have been approved by the Berlin Office of Health Protection and Technical Safety (G0218/17), with a yearly update of the experimental guidelines by the local authorities according to the 10 essential rules of ARRIVE [see latest update from July 14, 2020 and (Percie du et al., 2020)]. The experiments were performed in animals of either sex at an age of 51–76 weeks. The number of CAG repeats ranged from 182 to 201 and were determined by Laragen (Culver City, CA, United States). Care was taken that all experimental groups contained an equal number of males and females (±1). Apart from the body weight, no systematic differences could be detected in any of the evaluated indicators. Blinding was not applied, since the animals were used at a stage of HD when the experimenters could recognize the respective genotype and select the appropriate mice among a usually very small number of available animals. The necessary sample size was kept to a minimum and was calculated beforehand to achieve hypothesis testing at a significance level of 5%.

### Stereotaxic and Intravenous Injections of Viral Expression Vectors for Treatment and Diagnostic Purposes

Q175 WT and HET were anesthetized by intraperitoneal injection of a mixture containing 87.5 mg/kg ketamine and 12.5 mg/kg xylazine (both from Sigma-Aldrich, Taufkirchen) before receiving bilateral intrastriatal injections of PHP.eB-mRuby-gfaABS1D-EAAT2 (or respective isoforms/controls) as part of the treatment. The therapeutic vectors or controls were given at a concentration of 1.0*10^12^ gc/ml and an amount of 1 μl per site (1.0*10^9^ gc/striatum) at the following coordinates with respect to bregma (mm): anterior 0.75, lateral 2.0, ventral 2.5. The intravenous injections (100 μl) were performed under slight isoflurane anesthesia and adjusted to an injected amount of 2.0*10^11^–3.5*10^11^ gc/animal. The expression vector for the glutamate sensor AAV9-CamKII.iGlu*_*u*_*.WPRE-hGH (7.34*10^13^ gc/ml–0.3 μl) was applied at four sites at: anterior 1.5, lateral 1.56, 1.8, 2.04, 2.28, and ventral 1.7. The expression time between injection and sacrifice was 3–5 weeks.

### Validation of Transgene Expression

**FIGURE 1:**
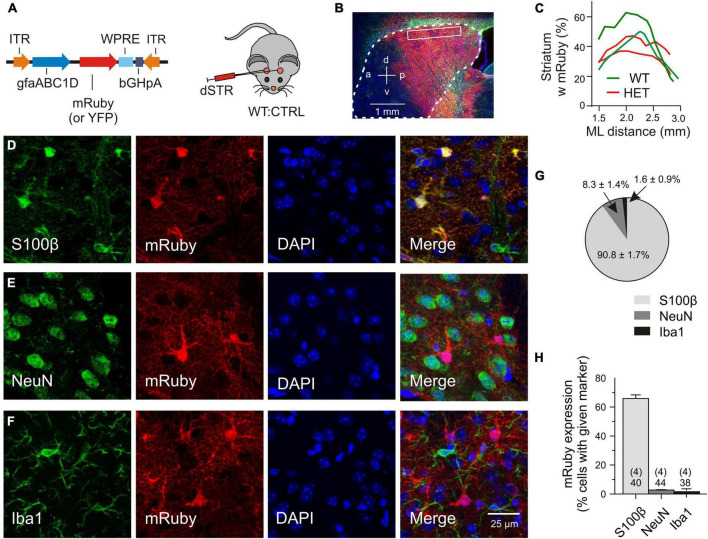
Validation of transgene expression. **(A)** Injected vector. **(B)** Transduced area (red) visualized with mRuby on the background of DAPI (blue) and GFAP immunofluorescence (green). The putative margin of the striatum is marked with a dotted line. **(C)** The mRuby+ area at the indicated mediolateral distance. **(D–F)** Immunostained sections for evaluation of viral expression patterns. Triple labeling with representative astrocytic **(D)**, neuronal **(E)**, or microglial **(F)** markers, together with an antibody against mRuby and nuclear counterstaining with DAPI. **(G)** Fraction of the astrocytic, neuronal or microglial phenotype among the mRuby+ population in the dorsal striatum. **(H)** Incidence of transduced cells within the S100β-, NeuN-, or Iba1-expressing cell populations. Numbers on columns: evaluated sections and animals (in brackets). bGHpA, bovine growth hormone polyadenylation sequence; DAPI, 4′,6-diamidino-2-phenylindol; dSTR, dorsal striatum; GFAP, glial fibrillar acidic protein; gfapABC1D, modified promoter sequence of GFAP; Iba1, ionized calcium-binding adapter molecule 1; ITR, inverted terminal repeat; mRuby, monometric Ruby; NeuN, neuron-specific protein, equivalent to Fox-3, S100β, S100 calcium-binding protein B; WPRE, woodchuck hepatitis virus post-transcriptional regulatory element; YFP, yellow fluorescent protein.

For the transfer of recombinant DNA the adeno-associated viral vectors were injected into the dorsal striatum, the region most affected by mHTT-related neurodegeneration (Vonsattel and DiFiglia, 1998). For detection of the transgene, mRuby or, in some experiments, mYFP was fused to the N-terminus of the *Slc1a2* sequence. Expression of the transgene was limited by the chosen experimental conditions, including (i) the amount of injected particles (a single injection of 1.0*10^9^ gc/striatum), (ii) the expression time (3–5 weeks, if not mentioned otherwise), (iii) the type of the viral serotype (PHP.eB) and (iv) the promoter sequence (gfaABC1D). To examine the abundance and specificity of transduction we have analyzed immunostained parasagittal sections of fixed brains after intrastriatal injection of mRuby CTRL or YFP CTRL vectors (Figure 1A). The outcome was similar (2 WT:CTRL and 2 HET:CTRL). In both cases cell counts were performed at a mediolateral distance of 2–2.5 mm within the boxed area of the dorsal striatum, each contributing 10 view fields of 100 μm × 100 μm. Figure 1B illustrates that the area of evaluation was well within the extensively labeled zone. The latter covered about 40% of the striatal volume (Figure 1C). There also was some labeling in the overlaying cortex and a small degree of unintended transgene expression in neurons and microglial cells. To examine the specificity of viral targeting to striatal astrocytes, the entire cell population in the dorsal striatum (boxed area in Figure 1B) was visualized with DAPI and tested for co-localization of mRuby with three cell-specific markers (Figures 1D–F). It was found that 90.8% of the transduced cells were S100β+ cells (astrocytes), 8.3% – NeuN+ cells (neurons) and 1.6% – Iba1+ cells (microglial cells) (Figure 1G). Considering the proposed astrocyte/neuron ratio of about 6:1 in the murine striatum (Chai et al., 2017), one can classify this transduction pattern as astrocyte-specific. We also determined the effective transduction rate of astrocytes. It amounted to 65.9 ± 2.47% (Figure 1H). In contrast, neurons and microglial cells were only transduced in 2.76 ± 0.44% and 1.64 ± 0.85% of the respective cell population. Together, these cell counts indicate that the viral transduction was local (dorsal striatum), specific (astrocytes) and effective (two thirds of the entire astrocyte population in the labeled area).

### Preparation of Acute Brain Slices

The animals were anesthetized with isoflurane, transcardially perfused with cooled aerated saline containing (in mM): NMDG – 92, KCl – 2.5, NaH_2_PO_4_ – 1.25, NaHCO_3_ – 25, glucose – 20, CaCl_2_ – 0.5, MgCl_2_ – 10, sodium pyruvate – 3, and sodium ascorbate – 5 (pH 7.35, 303 mosmol/l). After decapitation and removal of the brains, parasagittal (10 deg off) sections (300 μm) containing the striatum were prepared as previously described (Dvorzhak et al., 2016). The slices were kept in artificial cerebrospinal fluid (ACSF) containing (in mM): NaCl – 125, KCl – 3, NaH_2_PO_4_ – 1.25, NaHCO_3_ – 25, CaCl_2_ – 2, MgCl_2_ – 1, glucose – 10 (pH 7.3, 303 mosmol/l), supplemented with (in mM) sodium pyruvate – 0.5, sodium ascorbate – 2.8 and glutathione – 0.005. These perfusion and recovery solutions preserved the astrocytes better than physiological ACSF, sucrose- or choline-containing solutions, the criterion being the astrocyte resting membrane potential at break-in WT (≤−75 mV).

### Preparation of Striatal Lysates

The animals were anesthetized with isoflurane, transcardially perfused with cooled aerated saline containing (in mM): *N*-methyl-D-glucamine chloride (NMDG) – 92, KCl – 2.5, NaH_2_PO_4_ – 1.25, NaHCO_3_ – 25, glucose – 20, CaCl_2_ – 0.5, MgCl_2_ – 10, Na pyruvate – 3, and Na ascorbate – 5 (pH 7.35, 303 mosmol/l). If not mentioned otherwise, all chemicals and drugs mentioned here and in the following were obtained from Sigma-Aldrich (Taufkirchen). Brains were quickly removed, placed into a custom-made slicing mold and immersed into ice-cold oxygenated NMDG preparation solution. 2 mm thick sagittal slices where cut at a distance of 1–3 mm from midline. The striata were dissected and snap-frozen in liquid nitrogen. The tissue was pulverized under cryogenic conditions using a cryo-grinder set (CG 08-02, OPS Diagnostics, Lebanon, NJ, United States). The samples were solubilized in 200 μl lysis buffer containing (in mM) NaCl 150, Tris pH 7.5 50, *n*-ethylmaleimide 20, dithiothreitol (DTT) 1 supplemented with glycerol 5%, igepal ca-630 1% and Roche cOmplete protease inhibitor cocktail -1x (all from Sigma-Aldrich), kept on ice for 30 min and homogenized at 5,000 rpm using a Polytron PT1300D homogenizer. Cell debris was removed by centrifugation at 14.000 × *g* for 10 min at 4°C.

The cleared lysates were either directly submitted to WB analysis or used for the immunoprecipitation of YFP-tagged EAAT2 variants with magnetic beads for subsequent WB analysis or mass spectrometry.

### Western Blot Analysis of Striatal Lysates

Samples were prepared according to the NuPAGE Technical Guide of Invitrogen. Briefly, after denaturation in NuPAGE LDS sample buffer with DTT 50 mM for 10 min at 70°C the samples and markers were run on a Novex bis-tris gradient gel (4–12%, Thermo Fischer Scientific) using NuPAGE MOPS SDS running buffer and subsequently blotted on a Novex 0.45 μm nitrocellulose membrane (LC2001, Thermo Fischer Scientific). The membranes were washed and blocked in ReadyTector solution A (CANDOR Bioscience GmbH, Wangen im Allgäu), and the primary antibodies rabbit anti-EAAT2 1:2000 (Abcam, Cambridge, United Kingdom) and mouse anti-GAPDH 1:1000 (Sigma-Aldrich) were directly applied for 1 h at room temperature in solution B that also contained horseradish peroxidase (HRP) coupled to the secondary antibodies against rabbit or mouse, respectively. To detect mYFP, membranes were blocked for 30 min in 1× Roti-block solution (Carl Roth GmbH, Karlsruhe) before applying rabbit anti-GFP 1:1000 (ChromoTek GmbH, Martinsried) over night at 4°C in the same solution. Then the HRP-coupled secondary antibodies (Dianova GmbH, Hamburg) were applied at 1:2500 for 3 h at 4°C. Proteins were detected using respective kits from Biozym Scientific GmbH (Hessisch Oldendorf). Proteins were detected by chemiluminescence and submitted to image analysis with ImageJ.

### Immunoprecipitation of YFP-Tagged Excitatory Amino Acid Transporter 2 for Western Blotting or Mass Spectrometry

Striatal lysates were diluted with 300 μl wash buffer (in mM) NaCl 150, Tris pH 7.5 50, DTT 1, glycerol 5% and incubated for 2 h with 30 μl of pre-washed GFP-Trap magnetic agarose beads (gtma-20, ChromoTek, Planegg-Martinsried) using gentle rotation at 4°C. The beads-protein complexes were isolated using a DynaMag-2 magnet (Thermo Fisher Scientific), applying 3 wash-resuspension cycles before short-term storage of the immunoprecipitate at −18°C. The first wash solution contained igepal ca-630 0.05%. For WB analysis the beads were for 10 min incubated in the NuPAGE LDS sample buffer and treated as described in the previous chapter. For liquid chromatography tandem mass spectrometry (LC-MS), the beads were first incubated in digestion buffer (sodium deoxycholate – 1%, dithiothreitol – 10 mM, ammonium bicarbonate – 50 mM, 45 min, r. t.). The proteins were then submitted to alkylation with 55 mM chloroacetamide (30 min, r. t. complete darkness) and over-night digestion with 500 ng endopeptidase LysC (Wako, Neuss) and 500 ng sequence grade trypsin (Promega, Mannheim, GER) at 37°C. The samples were then acidified with formic acid (final concentration 1%).

### Excitatory Amino Acid Transporter 2 Interaction Proteomics

The peptides were extracted and desalted using the StageTips protocol. Separation was carried out using in-house-manufactured 20 cm fritless silica microcolumns with an inner diameter of 75 μm, packed with ReproSil-Pur C18-AQ 1.9 μm resin (Dr. Maisch GmbH, Ammerbuch), a 98 min gradient with a 250 nl/min flow rate of increasing Buffer B concentration (from 2 to 60%, Buffer B: 90% acetonitrile) on an High Performance Liquid Chromatography (HPLC) system from Thermo Fischer Scientific. The eluting peptides were directly ionized by electrospray ionization and transferred into a Thermo Orbitrap Fusion mass spectrometer. The instrument was operated in the data-dependent mode with performing full scans in Orbitrap (60K resolution; 4 × 10^5^ ion count target; maximum injection time 50 ms), followed by top 20 MS2 scans using higher-energy collision dissociation (NCE of 32; 15K resolution, 5 × 10^4^ ion count target; 0.7 m/z isolation window; maximum injection time: 250 ms). Only precursor with charge states between 2 and 7 were fragmented. Dynamic exclusion was set to 30 s. Raw data were analyzed using the MaxQuant software (v1.6.0.1). The internal Andromeda search engine was used to search MS2 spectra against a decoy UniProt database for mouse (MOUSE.2018-05), as well as the sequences of the mYFP fusion constructs, containing forward and reverse sequences. The search included variable modifications of oxidation (M) and N-terminal acetylation, deamidation (N and Q) and fixed modification of carbamidomethyl cysteine. Minimal peptide length was set to 7 amino acids and a maximum of two missed cleavages was allowed. The false discovery rate (FDR) was set to 0.05 for peptide and protein identifications. The integrated label-free quantification (LFQ) and the intensity-based absolute quantification (IBAQ) calculation algorithm were activated. Unique and razor peptides were considered for quantification. Retention times were recalibrated based on the built-in non-linear time-rescaling algorithm and MS/MS identifications were transferred between LC-MS/MS runs with the “Match between runs” option, in which the maximal retention time window was set to 0.7 min. The resulting text files were used for further analyses using the Perseus software package (omicX, v. 1.6.2.1). LFQ intensity values were used for quantification. Reverse hits, contaminants and proteins only identified by site were filtered out. Technical and biological replicates for each condition were defined as groups and intensity values were filtered for “minimum value of 3” per group. After log2 transformation missing values were imputed with random noise simulating the detection limit of the mass spectrometer. Imputed values are taken from a log normal distribution with 0.25× the standard deviation of the measured, logarithmized values, down-shifted by 1.8 standard deviations. The data was obtained from 4 test groups, each comprising at least 4 animals per group. The signals obtained from any given animal (pooling the tissue from both striata) were normalized to the mYFP signal median intensity calculated from all samples. Only proteins with >3 peptide detection and abundance of log2 intensities ≥ 23 in all 4 samples were included in the list of significant binders. Differential protein abundance was calculated using two-sample Student’s *t*-tests. Abundance differences between the samples with a *p*-value of ≤0.05 in a two-tailed *t*-test were considered significant. Please note that the present assay only included 3.790 from a total of 13.000 proteins so far identified in the mouse brain (Sharma et al., 2015).

The original mass spectrometry proteomics data has been deposited to the ProteomeXchange Consortium via the PRIDE partner repository with the dataset identifier PXD029194.

### Quantitative Immunofluorescence at Glutamatergic Synapses

Using deep isoflurane anesthesia, mice were transcardially perfused with 60 ml ice-cold phosphate-buffered saline (PBS) containing 4% (w/v) paraformaldehyde in PBS. Sagittal sections (30 μm) were prepared as previously described (Rothe et al., 2015). For the quantification of the transduction rate and transduction specificity freely floating sections were triple-stained with goat anti-td-Tomato (also detects RFP variants like mRuby) 1:6000 (Sicgen-Acris, Carcavelos), mouse anti S100β 1:2000 (Novus, Abingdon) and rabbit anti-Iba1 1:1000 (Wako Chemicals GmbH, Neuss) or double-stained with goat anti-td-Tomato and mouse anti-NeuN 1:500 (Merck Millipore, Darmstadt). Counter-staining for nuclei with 4′,6-diamidin-2-phenylindol (DAPI) 1:10000 was performed in both experiments. For the quantification of synaptic EAAT2 fluorescence, the sections were triple-stained with goat anti-tdTomato- 1:6000 (Sicgen-Acris), guinea pig anti-vGluT1 1:1600 (Synaptic Systems, Göttingen) and rabbit anti-EAAT2 1:2000 (Abcam), followed by respective secondary antibodies, as listed in Supplementary Table 1. All sections from the different test groups were stained together for reliable comparison of EAAT2 IF levels.

RGB 24 bit images (1,024 pixels × 1,024 pixels, no binning) were acquired from the dorsal striatum using a Leica TCS SP8 or DMI 6000 confocal microscope with an HCX PL Apo 63× oil objective (NA1.4) and stored in the tiff file format. Areas of interest (AOIs, 400 pixels × 400 pixels) were cropped from the larger view fields, selecting neuropil areas with a minimum of cell somata or vessels. Quantification of EAAT2 IF was performed using Image-Pro Plus (Media Cybernetics, Inc., Roper, Sarasota). For comparison purposes, the same staining conditions and acquisition settings were applied to achieve reliable EAAT2 quantification in 4–5 groups with at least 4 animals per group. Within the selected AOIs, smaller rectangular ROIs (40 pixels × 40 pixels, pixel size 90.19 nm) were centered to individual vGluT1+ spots to determine the level of synaptic EAAT2 IF. A threshold algorithm was used to define the boundaries of the EAAT2+ area excluding pixels with *F* < ROI mean + 0.5 SD. The data is expressed as integral intensity of suprathreshold pixels. The term “Synaptic integral EAAT2 IF” refers to the mean value from 30 individually assessed ROIs within the boundaries of one transduced (i.e., mRuby+) astrocyte.

### Sodium Imaging of Astrocytes in Striatal Slices

Our methods for sodium imaging in striatal astrocytes largely followed the techniques already described (Dvorzhak et al., 2016). Briefly, the slices were incubated for 20–30 min in oxygenated ACSF containing 222 μM of the membrane-permeable form of SBFI (#S-1264, Thermo Fisher Scientific), and 2.5% dimethyl sulfoxide (DMSO) and 0.5% Pluronic F-127 at 36°C. For recording of the L-aspartate-induced sodium transients, the gap junction blocker CBX (100 μM, Abcam) was added to the superfusion solution, along with blockers of ionotropic glutamate receptor blockers (DNQX 10 μM and MK801 1 μM, Tocris, Bristol, United Kingdom). Wide-field fluorescence imaging of SBFI-AM-stained slices was performed using a digital live acquisition imaging system (Andor Solis version 4.30.30034.0, Acal GmbH, Gröbenzell) and a sCMOS camera (Andor Zyla 4.2 plus) attached to an upright Zeiss microscope (Axio Examiner A1, Göttingen). Images were collected with a Zeiss 63× NA 1.0 water immersion plan apochromat objective. Cells were selected based on the resting levels of SBFI and mRuby fluorescence excited by a UVICO ultraviolet or visible light source (Rapp OptoElectronic, Hamburg), combined with suitable filter sets from Omega Optical (Brattleboro, VT, United States) attached to a FW 1,000 filter wheel (Applied Scientific Instrumentation, Eugene, OR, United States). Single wavelength sodium imaging was performed by excitation of SBFI at 380 nm (sodium-sensitive wavelength). SBFI emission was collected at >510 nm (dichroic mirror XF2002, emission filter XF3086, Omega Optical). Regions of interest (ROIs) with a size of 3.2 μm × 3.2 μm were defined on the cell body. Binning was 8 × 8. The spatial resolution was then 0.8 μm/pixel. The exposure times was set to 150 ms in all experiments. Images were acquired every 3 s. Custom-written software was used to control image acquisition and the valves operating the superfusion system. After 1 min of baseline recordings (20 images), L-aspartate (1 mM) was applied for 1 min followed by a 3 min washout period. Subsequently, the same routine was repeated in the presence of the glutamate transport blocker TFB-TBOA 2 μM (Tocris). For both traces the fluorescence change was calculated from the average of the baseline fluorescence intensities for each ROI as Δ*F*/*F* = (*F*_*SBFI*_ − *F*_*SBFI(baseline)*_/*F*_*SBFI(baseline)*_]. The difference between the two traces represents the L-aspartate-induced sodium transient that is mediated by all available glutamate transporters. The response to L-aspartate was verified by student’s t-test comparison between response peak and baseline.

### Glutamate Imaging at Single Corticostriatal Synapses

The methods established to image glutamate release from single corticostriatal presynaptic terminals and to identify synapses exhibiting an HD phenotype have already been described in some detail (Dvorzhak et al., 2019). Briefly, a vector for CaMKII-driven expression of the ultrafast glutamate sensor iGlu*_*u*_* was injected in the motor cortex (unilateral triple injections). Six weeks were allowed before sacrificing the animals. With this expression time the membrane-bound iGlu*_*u*_* sensor of corticostriatal terminals can even be detected at rest which helps the positioning of a stimulating micropipette for single bouton activation in the presence of action potential block. Acute slices were submerged into a perfusion chamber with a constant flow of oxygenated ACSF at a rate of 1–2 ml/min. The temperature during the recordings was maintained at 26–27°C. Single varicosities expressing the ultrafast glutamate sensor iGlu*_*u*_* (Helassa et al., 2018) were visualized using a Zeiss wide field microscope (Axioscope 2, FS Plus) with a 63×/NA 1.0 water immersion objective and brief (180 ms) discontinuous exposure to a 473 nm laser beam focused to a circular area of ∼4.5 μm in diameter. For evaluation of evoked responses, the iGlu*_*u*_* fluorescence was acquired at a frequency of 2.5 kHz from a rectangular ROI of 4 μm × 4 μm (20 pixels × 20 pixels, binning 2) using a sCMOS camera (Andor Zyla4.2 plus). Laser, camera and electrical stimulation of the axon/bouton were controlled by in-house written software routines. Each pixel of the ROI was evaluated separately. The iGlu*_*u*_* pixel signal was expressed as change of fluorescence intensity Δ*F* in % of baseline fluorescence of the given pixel. The baseline is the mean of the intensity values obtained during the 50 ms period prior to stimulation. For the construction of time- and space-dependent [Glu] profiles after evoked release, suprathreshold pixels were determined, the threshold being defined as 3 SD of the Δ*F*/*F* baseline. The stimulus-induced changes of suprathreshold Δ*F*/*F* in time or space will be referred to as “iGlu*_*u*_* transients” or simply “transients.” The term “Peak amplitude” refers to the peak Δ*F*/*F* value of an averaged intensity transient derived from all suprathreshold pixels. “Tau decay” or “TauD” is the time constant of decay derived by fitting a monoexponential function to the decay from the peak of the averaged transients. The spatial extension of the iGlu*_*u*_* signal is described on the basis of a virtual diameter derived from the area of all suprathreshold pixels combined to form a virtual circle. The area of suprathreshold pixels and the resulting virtual diameter were used as indicators of “Bouton size” (at rest, before stimulation) or “Spread” (after stimulation and glutamate release). The term “Peak spread” refers to the peak value of the averaged spread transient. Dysfunctional synapses could best be detected by analysis of single-pixel iGlu*_*u*_*, using the pixel with the highest iGlu*_*u*_* elevation at any given terminal. The highest iGlu*_*u*_* elevations were always found within or next to the bouton at rest. The peak amplitude of the single pixel transient with the highest iGlu*_*u*_* elevation are referred to as “Maximal amplitude.” The respective TauD values are referred to as “TauDmax.”

### Identification of Dysfunctional Synapses

To induce the glutamate release from individual synaptic boutons under physiological conditions, a depolarizing current pulse was applied through an ACSF-filled glass pipette (tip diameter <1 μM, resistance 10 MΩ) placed next to an axon in close proximity with a fluorescent varicosity. Responses were elicited at minimal intensity at a repetition frequency of 0.1 Hz. To challenge the glutamate uptake mechanisms at the site of release, individual synapses were stimulated under condition of blocked action potential generation (in the presence of tetrodotoxin, TTX, 1 μM and in elevated (5 mM) [Ca^2+^]_*ec*_. This standardized activation mode bypassed eventually existing disease-related differences in the myelination and excitability of corticostriatal axons. The identification of an HD phenotype in corticostriatal synapses is based on the TauD value of the glutamate transient (Dvorzhak et al., 2019).

### Behavioral Tests in Wild-Type and Q175 Heterozygote

On the day of sacrifice, the animals were submitted to a classical open field test (53 cm × 33 cm), as described in a previous study with Q175 homozygotes (Rothe et al., 2015). The mice were individually tested between 9 a.m. and 2 p.m. using an open-field box made of gray plastic with 53 cm × 33 cm surface and 19 cm walls, illuminated by a spot of 25 W placed 1 m above the shadow-free box. Monitoring of 5-min sessions was done by a video camera (Logitech C525 Webcam, 15 frames/s). A customary software was designed to quantify not only running activity but also the motor activity at rest. This required the calculation of the centroid of all pixels derived from the black mouse body. To this end, the background frame without mouse was subtracted from frames with mouse using an intensity threshold of mean + 3SD. A virtual mouse radius (v.m.r.) was derived from the area of suprathreshold mouse pixels approximated as a circle. The identification of the resting state is based on the time needed to cross 2 v.m.r. For more details and definitions see Supplementary Table 2.

For the “step-over test” the animal was placed into the center of a Petri dish (diameter 18.5 cm and wall height 2.8 cm). The movements were recorded with a video camera. Using offline analysis, a software routine determined the time between the take-off of the experimenter’s hand (in a black glove) and the moment when the animal has reached the outside of the dish with all 4 feet. This parameter was called “step-over latency” and correlated with the open field total path in 5 min.

### Data Evaluation and Statistical Analysis

The comparison of the means could be influenced by inter-animal variance. Therefore, in the case that individual sections, cells or synapses were obtained from different animals, multi-level (“nested data”) analysis was performed with Prism 8 (GraphPad, San Diego, CA, United States). *P*-values of <0.05 were considered statistically significant. Differences between the groups were tested with ANOVA or respective non-parametric methods (Kruskal–Wallis-test), followed by multiple comparison (Dunnet’s or Dunn’s tests). Significance levels were marked by asterisks, where * corresponds to *P* < 0.05, ^**^ – *P* < 0.01 and ^***^ – *P* < 0.001. The HET-CTRL data served as reference for comparison with WT-CTRL or HET-TEST. Effect strength was described according to Cohen’s *D* or Hedges’ *G*. *D*- or *G*-values larger 0.8 suggest that the respective effect was strong.

## Results

### Mutant Huntingtin-Induced Changes in the Striatal Excitatory Amino Acid Transporter 2 Interactome and Effects of C-Terminal Truncation

Failing glutamate uptake in mHTT-expressing mice could be derived from deficient EAAT2 expression and alterations in EAAT2 regulation. The latter should be reflected in the composition of the EAAT2 interactor spectrum. Previous studies have identified some of the EAAT2 binding proteins, see for instance (Bassan et al., 2008; Piniella et al., 2018). However, a complete overview of the EAAT2 interactors from native (whole animal) striatal tissue as well as information on their sensitivity to mHTT were still missing. To fill this important gap of knowledge we performed a mass spectroscopy analysis of immunoprecipitated transgenic EAAT2 and its C-terminal-truncated version EAAT2-S506X. This variant lacked the last 68 amino acids, i.e., it was devoid of almost the entire C-terminal domain. An YFP vector without the EAAT2 sequence served as CONTROL.

**FIGURE 2:**
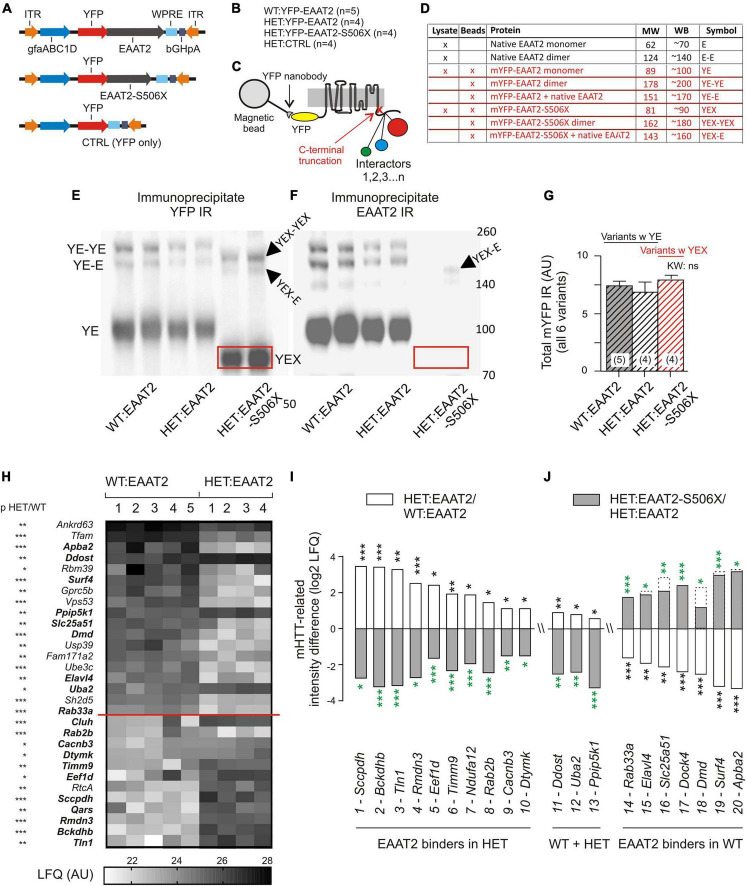
mHTT- and S506X-related changes in the abundance of YFP-EAAT2 binders. **(A)** Injected vectors. **(B)** Animal groups. **(C)** Scheme of immunoprecipitation experiment. **(D)** List of EAAT2 bands to be detected in the Western blots (WB) from the beads preparation (this figure) or lysates (Figure 3). **(E,F)** Western blot samples prepared from the beads YFP-immunoprecipitate. Note dimer bands and shift of the EAAT2-S506X band (YEX, boxed). **(G)** Similar amounts of mYFP immunoreactivity (YE or YEX bands) pulled down in the three animal test groups. **(H)** Heat-map plot illustrating the mHTT-related differences between WT:EAAT2 and HET:EAAT2. The signal from each animal was normalized to the mYFP median intensity value in a histogram constructed from all samples. The inter-group intensities were compared by a two-tailed *t*-test. The asterisks next to the listed genes denote the significance level of the difference. The proteins are listed with their gene names and sorted according to their abundance in WT:EAAT2. **(I,J)** Plot illustrating the recovery potential of significant EAAT2 binders. The LFQ intensity difference denotes the log2 difference between the compared groups. Compared were WT:EAAT2 vs. HET:EAAT2 (empty bars with black asterisks) and HET:EAAT2-S506X vs. HET:EAAT2 (filled bars with green asterisks). The dashed bars indicate the corresponding WT levels. An up-regulation is shown as upward bar, a down-regulation as downward bar. *p < 0.05, **p < 0.01, ***p < 0.001.

Wild-Type and HET mice received bilateral striatal injections with one of 3 YFP-tagged expression vectors (Figure 2A) and were sacrificed 3 weeks later. Figure 2B lists the resulting animal groups. Nanobody-coated magnetic beads were used to isolate the YFP-tagged EAAT2 protein and its interactors (Figure 2C). The quality of the immunoprecipitate was verified by YFP- and EAAT2-immunostained Western blots (WBs, see Figure 2D for identification and properties of the WB bands). Note the shift of the truncated YFP-EAAT2-S506X (boxed in red) in the respective WB bands (Figures 2E,F). The total amount of YFP pulled down by the beads was very similar in the 3 tested groups (Figure 2G), as should be the case if the amounts of “bait” were equal in the 3 groups, and the amount of “prey” (YFP-EAAT2) sufficed to saturate the binding sites.

The immunoprecipitate was further submitted to liquid chromatography tandem mass spectrometry (LC-MS). Statistical analysis revealed a total of 148 significant EAAT2 interactors in WT or HET. Our Supplementary Table 3: “Significant EAAT2-binders-in-WT-and-Q175HET.xlsx” lists these interactors along with some additional information on the binding characteristics. About 40% (58/148) were sensitive to mHTT, i.e., they exhibited a significant LFQ intensity difference between the EAAT2-expressing HET and WT (Table 1). But only few of them (*Tfam*, *Rbm39*, *Dmd, PDE10a*, and *Uba2*) were so far mentioned in connection with corticobasal pathologies. A differential effect of EAAT2 truncation (“X-effect”) was found in a total of 26 binders. Eighteen of them (69%) showed a protein down-regulation when HET mice expressed EAAT2-S506 instead of EAAT2.

**TABLE 1:** Summary binders with mHTT-related difference and effect of EAAT2 C-terminal truncation.

EAAT2 binders*				With mHTT effect**		With mHHT and X effect**	
Total	WT + HET	WT only	HET only	In HET down	In HET up	X effect up	X effect down
148	42	40	66	28	30	8 (31%)	18 (69%)

*FDR5, **t-test p < 0.05.

Figure 2H displays the individual LFQ intensity values of 30 EAAT2 binders with significant mHTT-related down- or up-regulation. They were sorted according to their mean LFQ intensity values in WT. The proteins of Figures 2I,J were selected to illustrate the X-effect in EAAT2 binders with FDR5 and listed according to their log2 LFQ intensity difference between the groups of HET:EAAT2 and WT:EAAT2 (empty bars/black asterisks). The log2 LFQ intensity difference for HET:EAAT2-S506X and HET:EAAT2 is shown as gray bars/green asterisks. Among the proteins up-regulated by mHTT (Figure 2I) were saccharopine dehydrogenase-like oxidoreductase (gene name *Sccpdh*), 2-oxoisovalerate dehydrogenase subunit beta (*Bckdhb*) and talin1 (*Tln1*) and NEDD8-activating enzyme E1 catalytic subunit (*Uba2*), also known as SUMO-activating enzyme subunit 2. A down-regulating X-effect is typically observed in HET with mHHT-related upregulation, whereas up-regulating X-effects were seen in WT with mHTT-related reduction of the protein abundance. Among the proteins most downregulated by mHTT were amyloid beta A4 precursor protein-binding family A member 2 (*Apba2*), surfeit locus protein 4 (*Surf4*) and dystrophin (*Dmd*). All 3 exhibited a recovery under the influence of EAAT2-S506x. Other proteins with mHTT-related down-regulation (e.g., phosphodiesterase 10A, *Pde10a*) remained unchanged after expression of EAAT2-S506X.

These proteomics results are consistent with the hypothesis that: (i) the EAAT2 interactome is influenced by the presence of mHTT, (ii) some of the mHTT-related changes are reversible and (iii) expression of C-terminal-truncated EAAT2 can block abnormal binders in favor of a more physiological protein interaction pattern. Together, these results both justify and motivate a more detailed investigation of the capacity of C-terminal-modified EAAT2 transgene expression to restore mHTT-impaired function.

### Mutant Huntingtin-Induced Changes in Native Excitatory Amino Acid Transporter 2 Levels and Effects of Excitatory Amino Acid Transporter 2-S506X

**FIGURE 3:**
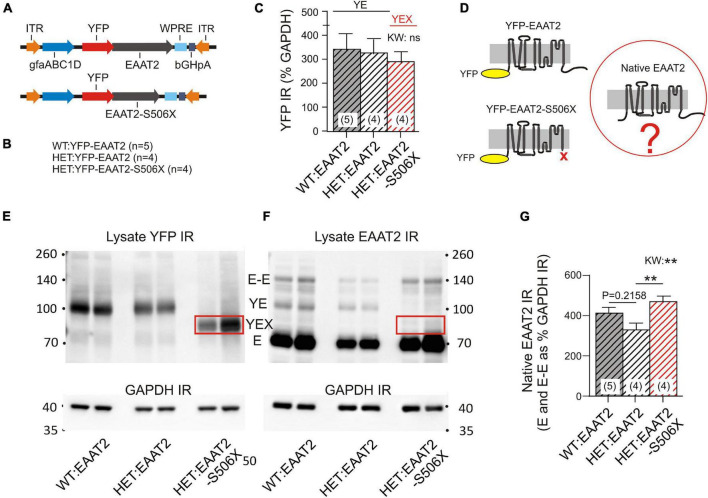
Effects of mHTT and S506X on the native murine EAAT2. **(A)** Injected vectors. **(B)** Animal groups. **(C)** Same amounts of YFP IR in the 3 samples. **(D)** Three principal variants of EAAT2 to be detected by in the lysates. **(E,F)** WB samples for quantification of YFP and EAAT2 IR in striatal lysates. At least four animals per group (shown are two). The boxed band YEX is not detected in **(F)** since the preferred antibody against EAAT2 binds to a region located on the deleted C-terminal. For the abbreviations see Figure 2D. **(G)** Mean intensity of native EAAT2 IR (mono- and dimers). Note increase of native EAAT2 level in HET:EAAT2-S506Xs. Non-parametric Kruskal–Wallis test (KW) and Dunn’s *post hoc* comparison. **p < 0.01.

Mass spectroscopy allowed a first glimpse at the normal and pathological binding partners of the EAAT2 transgene but it does not show what actually happened to the native murine EAAT2 protein. To provide the missing information, we analyzed the WBs of striatal lysates. WT and HET were injected with one of two EAAT2 vectors (Figure 3A) which resulted in the animal groups listed in Figure 3B. Quantification of the YFP immunoreactivity (IR) indicates that the YFP-tagged transgenes were equally expressed in the three animal groups (Figure 3C). However, the native murine EAAT2 devoid of YFP might be sensitive to transgene expression (Figure 3D).

The YFP-containing EAAT2 transgene has a higher molecular weight than the native EAAT2 (see Figure 2D for identification and properties of the WB bands). It should also be noted that the selected EAAT2 antibody recognizes only full-length EAAT2. Therefore the YFP-EAAT2-S506X band is missing in the EAAT2 WB (compare boxed in Figures 3E,F). Quantification of native EAAT2 IR revealed a significant increase in HET:EAAT2-S506X as compared to HET:EAAT2 (Figure 3G). As WT and HET differed in the occurrence of EAAT2 dimers, it was conceivable that the EAAT2-S506X-induced increase of native EAAT2 IR resulted from enhanced multimer formation. However, under the given experimental conditions, i.e., Western blotting, only a small percentage of EAAT2 (less than 15%) retained or re-established a dimeric form. The dimer-promoting effect of EAAT2-S506X, if at all present, remained below significance level.

These experiments lead to the interesting conclusion that the presence of an EAAT2 transgene may not be indifferent to native EAAT2. EAAT2-S506X expression in striatal astrocytes resulted in a higher abundance of native EAAT2.

### Mutant Huntingtin-Related Excitatory Amino Acid Transporter 2 Deficit at Immunostained Corticostriatal Synapses and Recovery in Heterozygote Treated With Excitatory Amino Acid Transporter 2-S506X or Excitatory Amino Acid Transporter 2-4KR

**FIGURE 4:**
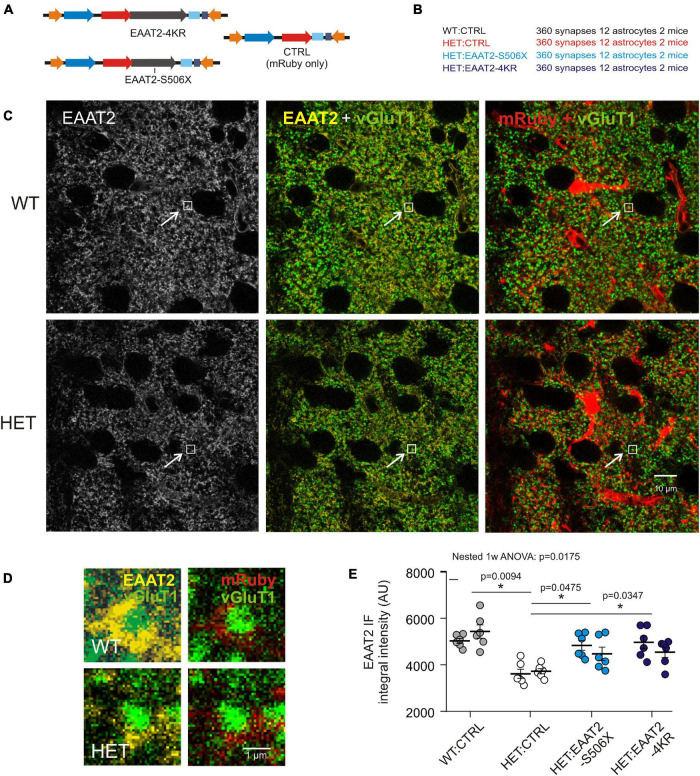
Evaluation of synaptic EAAT2 immunofluorescence in fixed sections. **(A)** Injected vectors. **(B)** Animal groups. **(C)** Confocal images from WT:CTRL and HET:CTRL to illustrate the mHTT-related decrease in the overall EAAT2 IF. In the illustrated samples the mean intensity values were 3078 ± 38.4 (WT:CTRL) and 2673 ± 31.7 (HET:CTRL). **(D)** Regions of interest (ROIs) comprising just one synapse. The ROIs correspond to the small boxed areas in **(C)**. The EAAT2-immunopositive area corresponds to an astrocyte perisynaptic process (PAP), as it is labeled with mRuby. **(E)** Quantification of integral EAAT2 IF from ROIs comprising only one vGluT1-immunopositive terminal. Numbers in brackets: evaluated synapses per group. Each data point is the mean value from 30 synapses on 1 mRuby-labeled astrocyte. Separate plot of data from the 2 animals per group. *p < 0.05 or 0.01.

**FIGURE 5:**
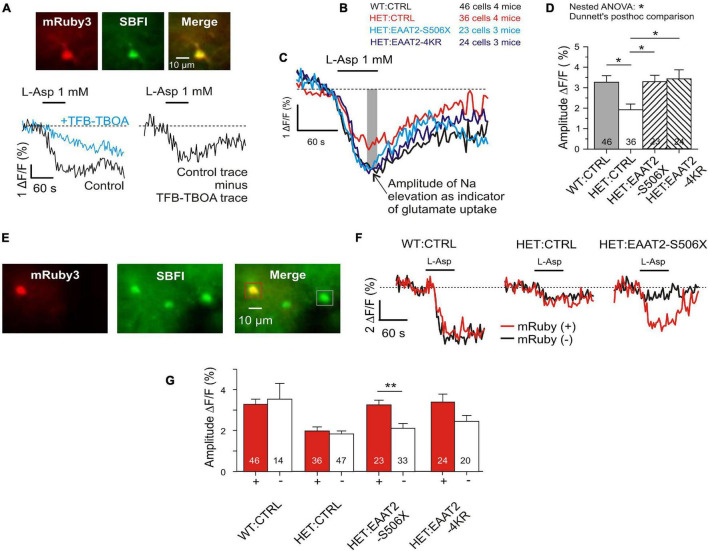
Recovery of glutamate uptake in striatal astrocytes. **(A)** Measurement of glutamate uptake by sodium imaging with SBFI, in the presence of CBX (100 μM), DNQX (10 μM), and MK801 (1 μM). Traces recorded in the absence and presence of TFB-TBOA (2 μM). The amplitude of this differential response was expressed as Δ*F*/*F* during the last 15 s of L-Asp application. *F* is the mean fluorescence at rest, before drug application. **(B)** Tested animal groups. **(C)** Averaged traces of SBFI fluorescence. Results from mRuby-positive astrocytes only. **(D)** Quantification of the L-aspartate-induced sodium elevation. Two-level (“nested”) statistics (animal level, cell level, and *post hoc* comparison between groups). **(E)** The applied SBFI loading protocol was selective for astrocytes. View field with 3 astrocytes, where one is transduced and two are not. **(F)** Traces from mRuby+ as opposed to mRuby– in three test groups. Averaged traces from one animal per group. **(G)** Comparison of results obtained in the different groups from mRuby+ vs. mRuby– astrocytes. *p < 0.05, **p < 0.01.

Examination of immunostained fixed brain sections could verify the above impression and answer the question of whether C-terminal-modified EAAT2 transgene can affect EAAT2 IR at sites where it really matters – the corticostriatal synapses. It should again be mentioned that the selected antibody only reacts against EAAT2 with intact C-terminal. Figures 4A,B lists the injected vectors and animal groups. Here (Figure 4) and in some of the following experimental series (Figure 5) HET mice were available in larger numbers. This provided us with an opportunity for the testing of more selective C-terminal modifications. In the case of HET:EAAT2-4KR, four C-terminal lysines (K) at position 518, 527, 551, and 571were replaced by arginine (R). It had been suggested that ubiquitylation of these C-terminal lysine residues mediates the interaction of the transporter with the endocytic machinery in a PKC-dependent manner (Gonzalez-Gonzalez et al., 2008) which may influence the availability of EAAT2 protein at synaptic sites.

The images obtained from the dorsal striatum of WT and HET (Figure 4C) illustrate, first of all, a noticeable shift toward lower EAAT2 immunofluorescence (IF) levels in HET, thereby confirming previous studies from non-injected HD mice (Estrada-Sanchez et al., 2009; Tong et al., 2014). Figure 4D shows the respective single synapse immunofluorescence (IF) in ROIs containing only one vGluT1-positive spot, i.e., one corticostriatal presynaptic terminal. The EAAT2-positive pixels co-localized with mRuby, i.e., they belonged to a perisynaptic astrocyte process (PAP). The graph of Figure 4E and Table 2 show the results of statistical evaluation of the EAAT2-immunofluorescent clusters within the analyzed ROIs. Both the HET:EAAT2-S506X and the HET:EAAT2-4KR groups differed from the HET:CTRL group, as their synaptic EAAT2 IF increased to nearly WT:CTRL level.

**TABLE 2:** Summary of experiments with functional testing.

mHTT-sensitive functional indicator	WT-CTRL				HET-CTRL				HET-EAAT2-S506X				Statistics			*Post hoc* MC HET-CTRL vs. HET-S506X		
	Mean	*SE*	N-c/t	N-a	Mean	*SE*	N-c/t	N-a	Mean	*SE*	N-c/t	N-a	Test	*F* (DFn, Dfd)	*P*	Test	*P*	Hedges’ G
#CAG repeats	nd				186.1	1.2		22	185.6	1.7		15				*t*-test	ns	
Body weight at sacrifice	31.28	0.86		21	25.35	0.77		22	26.41	1.08		15	ANOVA	0.29 (2, 55)	<0.0001	Tukey	ns	
**OF total distance traveled (m/5 min)**	18.72	**0.93**		**14**	**12.99**	**1.77**		**15**	**19.16**	**1.80**		**21**	**ANOVA**	**4.09 (2, 47)**	**0.0230**	**Tukey**	**0.027**	**−0.81**
**OF #starts/stops (in 5 min)**	**61.07**	**4.78**		**14**	**36.80**	**6.23**		**15**	**58.67**	**6.66**		**21**	**Kruskal–Wallis**	**4.17 (2, 47)**	**0.0216**	**B-K-Y**	**0.040**	**−0.78**
**Step-over latency (s)**	**56.7**	**10.00**		**18**	**179.1**	**46.6**		**19**	**51.6**	**9.8**		**14**	**Kruskal–Wallis**	**7.37 (2, 47)**	**0.0251**	**B-K-Y**	**0.041**	**0.79**
**Maximal/mean rest radius**	**2.53**	**0.04**		**14**	**2.81**	**0.11**		**15**	**2.58**	**0.21**		**21**	**ANOVA**	**4.64 (2, 47)**	**0.01**	**Tukey**	**0.038**	**0.75**
**Mean running velocity (mm/s)**	**99.53**	**5.14**		**14**	**81.57**	**6.04**		**15**	**109.45**	**8.06**		**21**	**ANOVA**	**4.15 (2, 26)**	**0.0220**	**Tukey**	**0.017**	**−0.87**
**Mean velocity at rest (mm/s)**	**37.39**	**1.78**		**14**	**26.48**	**3.69**		**15**	**36.91**	**3.12**		**21**	**ANOVA**	**3.72 (2, 47)**	**0.0318**	**Tukey**	**0.048**	**−0.70**
**Open field center time (%)**	**31.48**	**4.32**		**14**	**14.98**	**3.67**		**15**	**16.57**	**2.47**		**21**	**ANOVA**	**6.59 (2, 47)**	**0.0030**	Tukey	ns	
**Syn Glu transient spread (μ m)**	**1.23**	**0.05**	**48**	**14**	**1.45**	**0.08**	**46**	**21**	**0.94**	**0.08**	**29**	**8**	**Nested ANOVA**	**6.282 (2, 40)**	**0.0042**	**B-K-Y**	**0.0011**	**1.03**
**Syn Glu transient(max) TauD (ms)**	**3.03**	**0.28**	**48**	**14**	**8.21**	**1.39**	**46**	**21**	**2.57**	**0.31**	**28**	**8**	**Nested ANOVA**	**4.528 (2, 40)**	**0.0169**	**B-K-Y**	**0.012**	**0.76**
**Syn Glu transient(max) amp (DF/F%)**	**74.85**	**9.21**	**48**	**14**	**64.20**	**6.77**	**46**	**21**	**53.5**	**8.3**	**28**	**8**	**Nested ANOVA**	**0.31 (2, 40)**	**0.4751**	**B-K-Y**	**ns**	**0.24**
Syn EAAT2 IF total (a.u.)*	**5230**	**168.7**	**12/360**	**2**	**3663**	**81.42**	**12/360**	**2**	**4638**	**205.8**	**12/360**	**2**	**Nested ANOVA**	**12.25 (3,4)**	**0.0175**	**Dunnett**	**0.048**	**−1.87**
**SPN input resistance (MOhm)**	**114**	**10.3**	**18**	**8**	**165**	**17.0**	**14**	**8**	**127**	**13.5**	**8**	**5**	**Nested ANOVA**	**4.05 (2, 37)**	**0.0256**	B-K-Y	0.104	0.64
**ITonic(GABA) amp (pA)**	**11.33**	**2.37**	**13**	**6**	**2.99**	**1.03**	**14**	**8**	**5.78**	**3.37**	**7**	**5**	**Nested ANOVA**	**6.19 (2, 16)**	**0.0102**	B-K-Y	ns	

*Test includes 4KR group.

*MC, multiple comparison test; BKY, Benjamini, Krieger, Yekutieli test. In bold: Indicators with sensitivity to X-effect.*

These results support the idea that expression of C-terminal-modified EAAT2 can increase the availability of native EAAT2 at corticostriatal synapses. Moreover, elimination of four lysines may already be sufficient to rescue more than half of the lost native EAAT2 protein.

### Recovery of Astrocytic Glutamate Uptake

To validate the results from corticostriatal synapses in fixed sections and to obtain an estimate for the glutamate uptake capacity of individual astrocytes, a series of imaging experiments was performed in acute striatal slices loaded with the Na^+^ indicator SBFI-AM. Glutamate transport was elicited with L-aspartate. It is known that L-glutamate, L-aspartate, and D-aspartate are taken up with similar micromolar affinity (Arkhipova et al., 2019). In the present experiments L-aspartate was chosen for its physiological role as excitatory neurotransmitter (Morland et al., 2013) and its negligible effects at G-protein-coupled glutamate receptors. A major part of the L-aspartate-induced response but not the entire sodium signal was blocked by the high-affinity glutamate transport inhibitor TFB-TBOA (Figure 5A). Residual TFB-TBOA insensitive components are common and were attributed to additional effects on other transporters such as the Na, K-ATPase (Rimmele et al., 2017). Therefore only the amplitude of the TFB-TBOA-sensitive component of the Na^+^ elevation was used for quantification of the glutamate uptake activity. The first series of experiments compared four animal groups (Figure 5B). The traces of the averaged responses (Figure 5C) visualize the differences between WT and HET and the similarity of responses obtained from WT:CTRL, HET:EAAT2-S506X, and HET:EAAT2-4KR. Multilevel (nested) ANOVA confirmed a significant recovery of astrocytes transduced with EAAT2-S506X and EAAT2-4KR (Figure 5D and Table 2).

The present SBFI-loading protocol was adjusted to preferentially label astrocytes (Dvorzhak et al., 2016). This offered an opportunity to determine, in the same view field, the L-aspartate-induced glutamate uptake activity of transduced versus non-transduced astrocytes (Figures 5E,F). The difference between individual transduced and not-transduced astrocytes reached significance in HET:EAAT2-S506X (Figure 5G).

These results are in line with our quantification of native EAAT2 protein and show that in HET astrocytes not only C-terminal-truncated but also 4KR-edited EAAT2 can alleviate the mHTT-induced depression of glutamate uptake.

### Expression of Excitatory Amino Acid Transporter 2-S506X Promotes Glutamate Clearance at Corticostriatal Synapses

**FIGURE 6:**
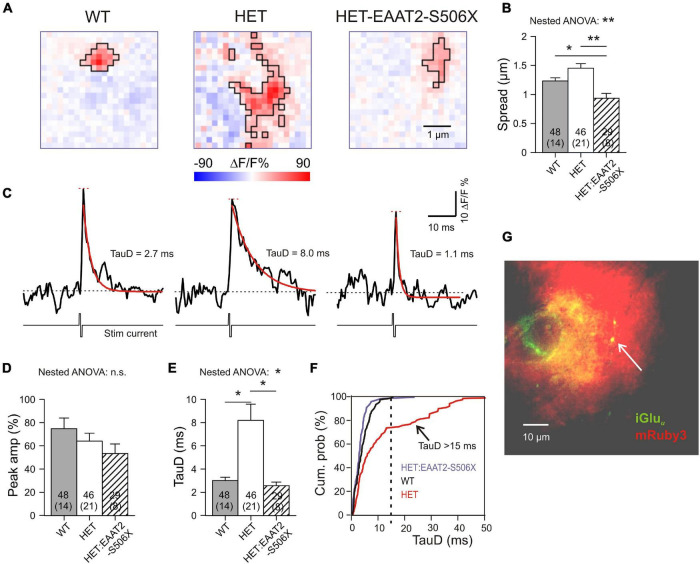
Single synapse glutamate clearance in the striatum after expression of EAAT2-S506X. **(A)** Representative examples from iGlu*_*u*_* imaging of single corticostriatal synaptic terminals in slices from WT, HET, and treated HET. Bilateral intracranial injections of HET with EAAT2-S506X. The selected images were acquired at the response peak to illustrate the extension of iGlu*_*u*_* elevation (boxed area). Pink pixels within the black boundaries are pixels where stimulation of the bouton elicited a fluorescence increase (Δ*F*) to values larger than the resting level prior to stimulation (*F*). **(B)** Glutamate spread at the ROI peak of the glutamate elevation (see Section “Materials and Methods” for definition of the parameter “Spread”). **(C)** iGlu*_*u*_* transients for the examples shown in **(A)**. Recordings before and after electrical stimulation of the given bouton in the presence of TTX. The traces represent the mean fluorescence intensity calculated from all suprathreshold pixels in the ROI. Red curves: Monoexponential functions fitted to the iGluu traces. In black – respective time constants of decay (TauD). **(D,E)** Quantification of results obtained from the pixel with the highest elevation of iGlu*_*u*_* fluorescence. **(F)** Cumulative probability plot to illustrate the absence of TauD values > 15 ms in WT and in EAAT2-S506X-treated HET. TauD values > 15 ms identify pathological synapses in HET. **(G)** Only synapses in the immediate vicinity of transduced astrocytes were included, as confirmed by the location of the tested iGluu-expressing varicosity on the territory of mRuby-positive astrocytes (arrowhead). *p < 0.05, **p < 0.05, n.s., not significant.

A previous publication from our lab (Dvorzhak et al., 2019) addressed the role of EAAT2 in single corticostriatal synapses by using CaMKII-driven expression of the genetically encoded “ultrafast” glutamate indicator iGlu*_*u*_* (Helassa et al., 2018). According to the previously established criteria, about 40% of corticostriatal synapses (19/46) in HET can be regarded as functionally impaired because glutamate clearance was significantly slower than in WT. But is this deficit due to a failure of astrocytic EAAT2? And if so, could a recovery of synaptic glutamate clearance be achieved by expression of truncated EAAT2? To answer both questions we now examined the effect of the EAAT2-S506X transgene in a set of 29 single corticostriatal synapses from 8 HET with manifest symptoms of hypokinesia. The present results from WT and HET were pooled with the previously characterized non-injected WT and HET synapses. The specimen recordings of Figure 6A illustrate our principal finding: Both in WT and in HET:EAAT2-S506X, the area of glutamate elevation is larger in HET. The quantification for the important parameter “Spread”(see section “Materials and Methods” for definition) is given in Figure 6B.

Further information on single synapse glutamate clearance was derived from evaluation of time-dependent changes of the iGlu*_*u*_* signal. The traces of Figure 6C represent the mean iGlu*_*u*_* intensity values (Δ*F*/*F* %) for the ROIs shown in Figure 5A. The recordings were taken in the presence of TTX before and after a short biphasic electrical stimulus via a glass pipette in the immediate vicinity of a labeled presynaptic terminal. Traces like these were used to extract two important indicators: “Peak amplitude” (short red horizontal line) and “Time constant of glutamate concentration decay” (TauD, see values next to the red monoexponential fitting curve). Similar traces were constructed from single pixels. The transients derived from the pixel with the highest fluorescence increase reflect the release and clearance of glutamate next to the presynaptic active zone, i.e., the site of vesicle exocytosis. It was found that TauD, but not peak amplitude, were affected by the expression of EAAT2-S506X (Figures 6D,E and Table 2). In contrast to untreated HET, synapses with prolonged decay (>15 ms) were never encountered in HET:EAAT2-S506X (Figure 6F). It should be pointed out that the present single synapse data has exclusively been collected from terminals located on the territory of transduced astrocytes. The arrowhead in Figure 6G points to a bright varicosity on the background of the red fluorescent area generated by the dendritic field of one mRuby-expressing astrocyte.

These results (i) verify the contribution of astrocytic EAAT2 to the corticostriatal glutamate clearance and (ii) demonstrate the capacity of corticostriatal synapses to return to normal performance. C-terminal EAAT2 truncation accelerated glutamate uptake and reduced its spread.

### Recovery of Motor Performance in Treated Q175 Heterozygote

Starting with an age of 10–12 months Q175 HET exhibit symptoms of hypokinesia. Thus, by the time of testing (14–18 months) mHTT-expressing neurons and astrocytes must have undergone progressive changes for several months. The aim of the following experiments was to quantify the mHTT-induced alterations in the locomotion and to find out to what extent these alterations could still be influenced. To gain a set of easy-to-quantify indicators of hypokinesia, the mice were submitted to two video-recorded tests: (i) the step-over latency test, SOLT (Dvorzhak et al., 2019) and (ii) the open field test, OFT (Menalled et al., 2012; Rothe et al., 2015). The tests were performed immediately before the viral vector injection and 3–6 weeks later, before animal sacrifice. The compared animal groups were WT:CTRL, HET:CTRL and HET:EAAT2-S506X. All animals were carefully matched according to CAG repeat numbers and age.

**FIGURE 7:**
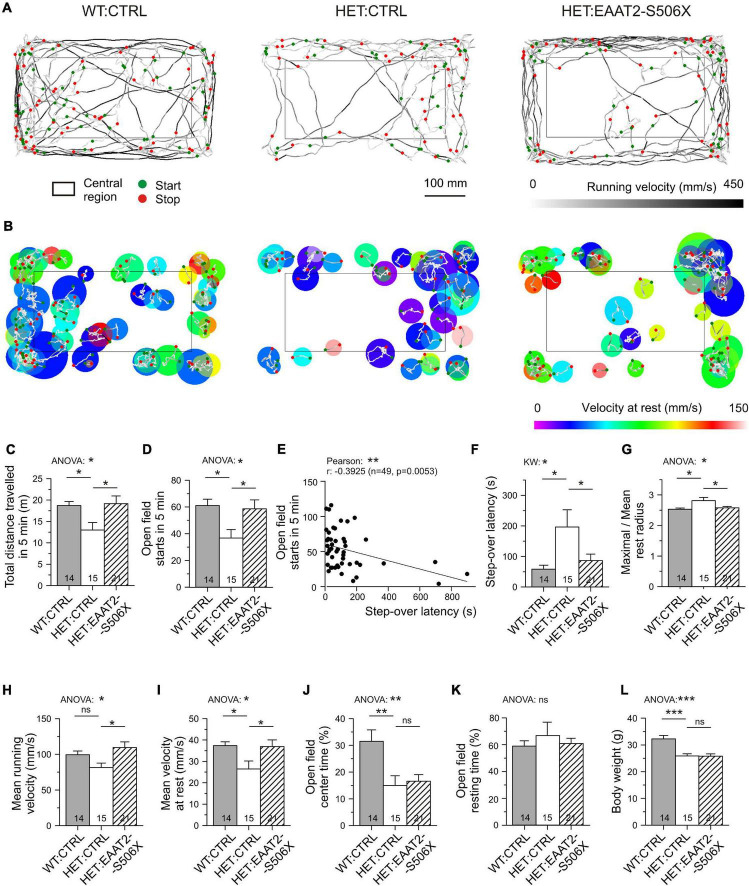
Effects of bilateral EAAT2-S506X expression on the motor performance of Q175 mice. **(A)** Recordings of open field trajectories showing the mean running velocity between starts and stops (green or red circles, respectively). Gray level coding of instantaneous running velocity. **(B)** Motor activity at rest. Color coding of movement velocity at rest. **(C,D,F–K)** Quantification of results from open field testing. **(E,F)** Step-over latency – inverse relationship with number of starts in the open field, and sensitivity of step-over latency to mHTT and treatment. **(L)** Matched age-, CAG-, and body-weight composition of the test groups (only body weight illustrated). *p < 0.05, **p < 0.01, ***p < 0.001.

The behavioral examination provided us with a set of six criteria to classify an animal or animal group as being hypokinetic or recovered from hypokinesia. Specifically, the step-over latency of SOLT was regarded as a direct measure of the time needed to initiate exploration in the open field. Its usefulness was supported by a larger number of observations from non-injected HD mice (Supplementary Figure 1). Step-over latencies longer than 300 ms were almost exclusively found in HET which could contribute to the detection of recovery effects. The other indicators are based on a more complex analysis of the movement trajectories in the open field (Figures 7A,B, see Supplementary Table 2 for definitions of the analyzed parameters). Five out of 6 movement parameters extracted from OFT were sensitive to the C-terminal-modified EAAT2. The most commonly used OFT indicator “total distance traveled in 5 min” was found to be decreased in HET:CTRL and recovered in HET:EAAT2-S506X if compared to WT:CTRL (Figures 7A,C).

The incidence of starts from rest is possibly the most reliable indicator of depressed movement initiation (Figures 7A,D). It is inversely correlated with the step-over latency (Figure 7E). After treatment with EAAT2-S506X both parameters exhibited changes toward WT:CTRL levels (Figures 7D,F). Also noteworthy is the treatment-related reduction of the maximal to mean radius of the resting area, i.e., the extension of the resting area until a new movement starts (Figures 7B,G). One could further expect an mHTT-related alteration in the velocity of body displacement, but it is not known whether this would equally apply to rest and running. It turned out, that EAAT2-S506X affects movement velocity both during the resting and running phases. Moreover, the direction of the changes treatment were similar and showed recovery with EAAT2-S506X (Figures 7H,I).

Other aspects of open field behavior were unchanged by viral treatment, such as the relative time spent in the open field center, or the open field resting time (Figures 7J,K) which suggests that these indicators might not be related to the site of injection, the dorsal striatum, or not be representative of exploratory motor activity. The typical reduction in the body weight remained as well (Figure 7L).

Together, these experiments present another strong argument in support of the hypothesis that molecular uncoupling of EAAT2 from its intraastrocytic interaction partners can alleviate mHTT-induced symptoms even at advanced stages of the disease. The results also underscore the relevance of striatal astrocytes for the initiation and speed of exploratory movements.

### Over-Expression of Full-Length *Slc1a2* Is Not Sufficient to Alleviate the Symptoms of Hypokinesia

**FIGURE 8:**
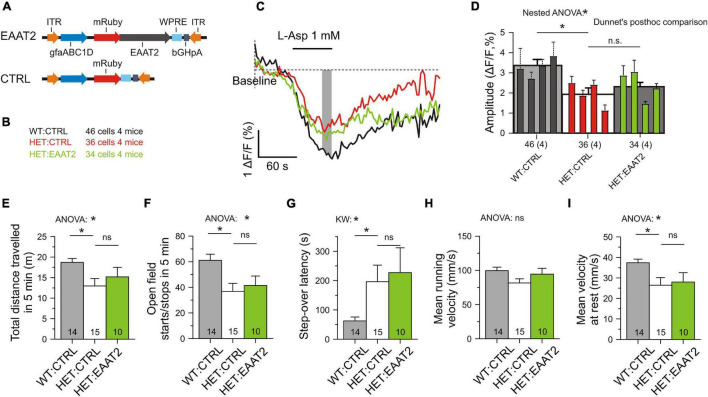
No recovery of astrocytic glutamate uptake and motor performance in mice overexpressing the full-length form of EAAT2. **(A)** Injected vectors. **(B)** Animal groups. The injected animals were matched for age, body-weight and the number of CAG-repeats. The numbers refer to the experiments with SBFI imaging. **(C)** Measurement of single-cell glutamate uptake by sodium imaging with SBFI. Same conditions as in Figure 5. Averaged traces from mRuby3+ astrocytes only. **(D)** Quantification of results with two-level (“nested”) ANOVA statistics (animal level and cell level) and two-level *post hoc* tests for the differences between the test groups. **(E–I)** Lack of recovery from the signs of hypokinesia. Here as in all other figures: n.s., not significant. *p < 0.05.

Finally we wanted to clarify the performance of mice with a full-length EAAT2 transgene (Figures 8A,B). SBFI imaging was again performed in transduced astrocytes, but a recovery of glutamate uptake was not observed (Figures 8C,D). Moreover, comparison of the L-aspartate-induced Na transients in HET:CTRL, HET:EAAT2-S506X, and HET:EAAT2 showed that EAAT2-S506X-treated mice outperformed (Kruskal–Wallis test: *P* = 0.0004) not only with respect to HET:CTRL (Dunn’s test: *P* = 0.0004) but also with respect to HET:EAAT2 (Dunn’s test: *P* = 0.0052). The respective mean, SE and N of Δ*F*/*F* (%) were for HET:CTRL: 2.01 ± 0.18 (*N* = 36), HET-EAAT2: S506X: 3.28 ± 0.23 (*N* = 23), HET:EAAT2: 2.31 ± 0.23 (*N* = 34). Locomotion did not recover either (Figures 8E–I). The comparison of HET:EAAT2 with HET:EAAT2-S506X-treated mice showed that the latter outperformed (ANOVA *p* < 0.05) with regard to the number of open field starts/stops in 5 min and the mean running velocity (mm/s). The respective mean, SE and N were for HET:CTRL: 36.80 ± 6.23 and 81.57 ± 6.04 (*N* = 15), HET:EAAT2-S506X: 58.67 ± 6.66 and 109.45 ± 8.06 (*N* = 21), HET:EAAT2: 41.40 ± 7.38 and 94.45 ± 8.57 (*N* = 10).

We concluded that over-expression of full-length EAAT2 is not sufficient to rescue astrocytic glutamate uptake in the striatum and it does not ease the symptoms of hypokinesia.

### Supplemental Experiments

A series of supplemental experiments addressed the efficacy of EAAT2-S506X treatment with systemically applied vectors (Supplementary Figure 2) and the influence on neuronal excitability (Supplementary Figure 3). In both cases the effects of S506X remained below the significance level required with nested ANOVA.

## Discussion

The results underscore the importance of C-terminal-dependent EAAT2 molecular interactions and demonstrate that mHTT-related deficits can be reversed by the expression of a C-terminal-modified EAAT2 transgene. The latter conclusion is based on tests for changes in synaptic glutamate clearance, astrocytic glutamate uptake and self-induced locomotion.

### What Could Be the Cause of Failing Glutamate Uptake and a Molecular Basis of Partial Recovery?

In principle, insufficient glutamate uptake could be due to changes in the relative abundance of functional EAAT2 isoforms (Goursaud et al., 2011) or aberrant RNA splicing (Lin et al., 1998; Scott et al., 2011). At least 3 EAAT2 variants are known to differ in their C-terminal sequence: EAAT2a, EAAT2b, and EAAT2c [GLT-1a, GLT-1b and GLT1c in rodents, see Vandenberg and Ryan (2013) for further information]. In the rat brain, EAAT2a is ∼15-fold more abundant than EAAT2b (Holmseth et al., 2009). Both are found at glutamatergic synapses (Al Awabdh et al., 2016), but only EAAT2b is required for the regulated as opposed to constitutive glutamate transport (Underhill et al., 2015). The C-terminus of EAAT2b but not EAAT2a contains a sequence predicted to interact with PDZ-containing proteins, including PICK1 (Wheatley et al., 1992; Bassan et al., 2008), PSD95 (Gonzalez-Gonzalez et al., 2009), or DLG1 (Underhill et al., 2015). By interaction with these proteins, EAAT2 can increase its internalization and/or lateral mobility in the astrocyte membrane due to activation of CamKII (Underhill et al., 2015) or high concentration of glutamate (Al Awabdh et al., 2016). It was shown that experimental immobilization of EAAT2 prolongs the postsynaptic synaptic currents (Murphy-Royal et al., 2015). Given that the duration of the glutamate transport cycle [∼12 ms at Schaffer collateral synapses (Bergles and Jahr, 1998)] is relatively long in comparison with the glutamate receptor binding, it was hypothesized that lateral movement of the substrate-transporter complex and a rapid membrane turnover to replenish fresh unbound transporter molecules are requirements for effective glutamate buffering at synaptic sites. To ensure the high abundance of transporter molecules around a glutamate-releasing terminal (Lehre and Danbolt, 1998) the turnover of the transporter from the cytoplasm into the membrane should also be high. However, how the degradation/replenishment of EAAT2 protein at a tripartite synapse actually works is not yet clear.

The present experiments in symptomatic Q175 HET are, to the best of our knowledge, the first attempt to characterize an HD phenotype in the EAAT2 interactome by using immunoprecipitation of mYFP-tagged native and C-terminal-truncated EAAT2 as “bait.” The focus was on alterations produced by mHTT, on one side, and changes due to C-terminal truncation of EAAT2, on the other side. It became quite obvious that the presence of mHTT produced major changes in the abundance of full-length EAAT2 binding partners, while expression of EAAT2-S506X resulted in a partial or even full return to the interaction pattern seen in WT.

The changes in HET included the disappearance of normally existing, potentially necessary interactors, such as dystrophin or PDE10a, and the appearance of new, potentially pathological interactors, such as the proteins encoded by *Tln1*, *Rab2b*, *Cacnb3*, or *UBA2*. Dystrophin is quite plentiful in astrocytes, especially at the endfeet, and it has been shown that dystrophin-glycoprotein complexes provide a link between laminin and the cytoskeleton (Sato et al., 2018) thereby contributing to the stabilization of aquaporin4 and Kir4.1 in particular subregions of the astrocyte plasma membrane (Enger et al., 2012). The function of dystrophin at glutamatergic corticostriatal synapses has not yet been explored, but there is evidence for a role of a dystrophin-associated protein complex in the pathogenesis of parkinsonian symptoms (Spuler et al., 2010). That PDE10a belongs to the astrocytic interactor proteins of EAAT2 is new information, but it has been reported that loss of PDE10a could be an important early biomarker of human HD (Ahmad et al., 2014; Russell et al., 2014) and has been considered as a potential target for molecular intervention (Beaumont et al., 2016). While our results confirmed the mHTT-related loss of PDE10a, we found no evidence for C-terminal-related recovery which points to other sites of interaction.

Of particular interest are the up-regulated proteins and their sensitivity to treatment with EAAT2-S506X. Talin1 links integrins to the actin filaments of the cytoskeleton and thereby contributes to the first steps of local cell adhesion (Campbell and Ginsberg, 2004). Its role in astrocytes and/or HD is unknown but its plasma titer is reported to increase during active phases of multiple sclerosis (Muto et al., 2017). Iqsec3 belongs to the group of guanine exchange factors (GEFs) and is implicated in synapse assembly. An up-regulation of Iqsec3 was implicated in the formation of inhibitory synapses (Kim et al., 2020) which could be part of a compensatory response in HET.

Concerning Uba2, it is known that UBA2 and SAE1 form a heterodimer that functions as a SUMO-activating enzyme. Sumoylation – the covalent attachment of SUMO1 to lysine residues – is a posttranslational modification process with relevance to HD (Steffan et al., 2004). Interestingly, non-sumoylated EAAT2 resides in the plasma membrane while the product of SUMO fusion with EAAT2 tends to form aggregates in the cytoplasm (Foran et al., 2011). When comparing the UBA2 levels precipitated by EAAT2-S506X vs. EAAT2 in HET, it was apparent that the loss of C-terminal interaction motives largely prevented the interaction between EAAT2 and UBA2. In cultured astrocytes, a proteolytically released C-terminal fragment of EAAT2 modified by SUMO1 was also found in the nucleus, with inhibitory effects on EAAT2 transcription (Gibb et al., 2007; Foran et al., 2011). The relevance of this EAAT2-derived signaling mechanism was demonstrated *in vivo*, in a mutant SOD1 mouse model of amyotrophic lateral sclerosis (ALS), where knock-in of a modified *Slc1a2* isoform with a defective caspase-3 cleavage site prolonged the life span of mice afflicted by the disease (Rosenblum et al., 2017).

It is interesting that the present evaluation of native EAAT2 protein at glutamatergic synapses and glutamate uptake in striatal astrocytes rendered almost equal effects of EAAT2-4KR and EAAT2-S5096X. This result underlines the critical role of C-terminal lysine-mediated interactions such as sumoylation and ubiquitination in the regulation of glutamate uptake. It should also be noted that there is a basal constitutive internalization of the transporter, with a critical role of the lysines 497, 517, 526, 550, 558, 570, and 573 NCBI Reference Sequence: XP_030104754.1 at the EAAT2 C-terminal (Gonzalez-Gonzalez et al., 2008; Martinez-Villarreal et al., 2012). Here we mutated only the lysine residues with the most prominent effect on transporter internalization also considering their shifted position in the human EAAT2 sequence, i.e., 518, 527, 551, and 571.

In the context of previous reports on the constitutive internalization of EAAT2 and the toxic effects of sumoylated C-terminal EAAT2 cleavage products, it is tempting to propose that an astrocyte expressing *mHTT* presents with largely altered conditions for both transcriptional control and protein sorting, which perhaps explains why in Q175 HET a mere stimulation of full-length EAAT2 failed to eliminate the deficits in the glutamate uptake and motor performance of HET:EAAT2. Of course, much more work is needed to fully unravel the molecular determinants of EAAT2 function in the striatum and other regions of the brain.

### Which Cellular Markers Report Failure or Rescue of Glutamate Clearance?

In the past glutamate uptake has mostly been quantified on the basis of tritium-labeled striatal tissue or synaptosomal preparations (see Section “Introduction”). In comparison with the measurement of tissue glutamate uptake, SBFI imaging of individual fluorescence-tagged astrocytes offers the advantage that the uptake activity can directly be compared in transduced *vs.* non-transduced astrocytes. In this way the results should be less affected by other mHTT-induced alterations, such as changes in the size and density of astroglia in the striatum. Our present analysis provides the still missing proof that the deficits in locomotion and synaptic glutamate clearance were in fact associated with: (i) signals mediated via the EAAT2-C-terminal, as opposed to other EAAT2 domains and (ii) striatal astrocytes as opposed to other cell types/regions in the mouse brain.

Unfortunately, the subcellular resolution of glutamate uptake with sodium imaging is not very high, even in the case of focal glutamate uncaging (Dvorzhak et al., 2016). This limits the use of SBFI imaging for the estimation of the glutamate clearance at the sites where it matters – the tripartite synapse. Previous attempts to reveal mHTT-related clearance deficits with glutamate sensors (Parsons et al., 2016; Parievsky et al., 2017) also failed, most likely due to methodical limitations (slow glutamate sensors, non-selective general tissue depolarization, low temporal and spatial resolution of synaptic responses). Considering, however, the importance of the corticostriatal pathway for the initiation of self-induced movements (Plotkin and Surmeier, 2015) it was found worthwhile to establish a new approach for the evaluation of the glutamate transients at individual glutamatergic synapses in the dorsal striatum (Dvorzhak and Grantyn, 2020). The most telling indicators of clearance deficiency were the time constant of decay and the spread of the stimulus-induced glutamate elevation as reported by the ultrafast glutamate sensor iGlu*_*u*_* (Helassa et al., 2018; Dvorzhak et al., 2019). It was already known that the duration of corticostriatal EPSCs increased in HD mice (Dvorzhak et al., 2019) and, respectively, decreased after the exposure to pharmacological blockers of glutamate transport or modulators of NF-κB-dependent transcription (Lee et al., 2008; Ghosh et al., 2016). A faster decay of the glutamate elevation at corticostriatal terminals located on S506X-expressing astrocytes can therefore be regarded as the currently most convincing argument in support of the astrocytic contribution to the glutamate clearance in mice expressing a mutant form of huntingtin.

### Is Region- and Cell-Type-Restricted Expression of Artificial Excitatory Amino Acid Transporter 2 Variants a Useful Approach to Study Disease-Related Pathology?

Huntington’s disease is a severe inherited neurological disorder with diverse clinical symptoms and variable onset. With a respective gene test it can be diagnosed much before the onset of motor or other symptoms. Great efforts are therefore being made to reduce the expression of *mHTT* inside and outside the central nervous system before the disease actually starts (Mrzljak and Munoz-Sanjuan, 2015; Tabrizi et al., 2019). *mHTT-*lowering therapies have reached an advanced stage of preclinical testing or even entered the phase of first clinical trials (Mullard, 2019). In animal models of HD, the most effective approaches include the use of self-inactivating KamiCas9 system for editing the huntingtin gene (Merienne et al., 2017), injection of *mHTT* transcription-targeting viral vectors (Zeitler et al., 2019) and application of siRNAs, shRNAs, or miRNAs for the reduction of *mHTT* mRNA and protein level (see Kaemmerer and Grondin, 2019 for review).

It is obvious that gene therapy is becoming more problematic at symptomatic stages of the disease. In HD, as well as other neurodegenerative disorders, late-stage pathology may include numerous compensatory mechanisms which would make it increasingly difficult to implement a causal therapy. But substantial therapeutic benefits may still be possible, notably if basic research can reveal a (virtual) bypass in the signal flow connecting the elements of molecular intervention and motor outcome. In the present experiments with mHTT-expressing Q175 mice, a robust motor response was induced by local (striatum), cell-selective (astrocytes) and site-directed (EAAT2 C-terminus) transgene expression. Such result is to some extent surprising because most evidence-based models describe the initiation of voluntary movements as the result of a multi-level parallel and distributed information processing in a large number of brain structures [see for instance (Morita, 2014)]. That a particular cell population can effectively control a set of motor acts has mostly been implicated in “lower” vertebrates or invertebrates. However, reports on specific links between motor performance and specific transgene expression are already accumulating [see, for instance, Nagai et al. (2019); Percie du et al. (2020)] and may help to identify new signaling pathways.

It is interesting and important that not all symptoms of HD were sensitive to the expression of modified EAAT2. There was, for instance, no change in the body weight or in the open field center time. This might be regarded as one more argument in support of the idea that the modification of EAAT2 in striatal astrocytes preferentially affects the performance of the synapses.

The present results have promise with regard to small molecule therapy of neurological conditions with hypokinesia, including HD, Parkinson’s disease, stroke and toxin-induced brain damage. These disorders may share similar alterations in the regulation of synaptic glutamate transport. We therefore consider it highly interesting that the interruption of C-terminal-dependent regulation of EAAT2 renders beneficial effects at both the cellular and behavioral level. Blocking pathological signals might be a good strategy where causal therapy is difficult to achieve.

## Data Availability Statement

The data presented in the study are deposited in the PRIDE repository, accession number PXD029194.

## Ethics Statement

The animal study was reviewed and approved by Berlin Office of Health Protection and Technical Safety (G0218/17).

## Author Contributions

SH prepared and injected the vectors, validated the transgene expression, and contributed to the mass spectrometry, Western blotting, sodium imaging, and behavioral experiments. AD performed the single synapse imaging and behavioral data analysis and contributed to the sodium imaging experiments. MK and RG accomplished the proteomics analysis. SR-N performed patch-clamp recordings and astrocyte imaging. SA performed immunostaining and confocal microscopy. All authors listed have made a substantial, direct, and intellectual contribution to the work, and approved it for publication.

## Conflict of Interest

The authors declare that the research was conducted in the absence of any commercial or financial relationships that could be construed as a potential conflict of interest.

## Publisher’s Note

All claims expressed in this article are solely those of the authors and do not necessarily represent those of their affiliated organizations, or those of the publisher, the editors and the reviewers. Any product that may be evaluated in this article, or claim that may be made by its manufacturer, is not guaranteed or endorsed by the publisher.
