# Supplementary material for: Uncoupling the Excitatory Amino Acid Transporter 2 From Its C-Terminal Interactome Restores Synaptic Glutamate Clearance at Corticostriatal Synapses and Alleviates Mutant Huntingtin-Induced Hypokinesia
Source: Front Cell Neurosci. 2022 Jan 31;15:792652. doi: 10.3389/fncel.2021.792652 (PMC8841566; doi:10.3389/fncel.2021.792652)
Supplement: Supplementary file 1 [file Data_Sheet_1.docx]

**SUPPLEMENTAL MATERIALS**

| **Supplemental Table 1.** Key resources.   \| **RESOURCE** \| **SOURCE** \| **IDENTIFIER** \| \| --- \| --- \| --- \| \| ***Animals*** \|  \|  \| \| Z-Q175-KI \| Jackson Labs, Maine, USA \| Stock# 027410 \| \| ***Antibodies*** \|  \|  \| \| Donkey anti-Goat IgG, Alexa Fluor® 555 (1:800) \| Life Technologies, Darmstadt, GER \| Catalogue #A-21432 RRID: AB_2535853 \| \| Donkey anti-Guinea pig IgG, Cy™5 (1:200) \| Jackson ImmunoResearch, Biozol, Eching, GER \| Catalogue #706-175-148 RRID: AB_2340462 \| \| Donkey anti-Mouse IgG, Alexa Fluor® 488 (1:800) \| Life Technologies, Darmstadt, GER \| Catalogue #A-21202 RRID: AB_141607 \| \| Donkey anti-Rabbit IgG, Alexa Fluor® 488 (1:800) \| Life Technologies, Darmstadt, GER \| Catalogue #A-21206 RRID: AB_141708 \| \| Donkey anti-Rabbit IgG, Alexa Fluor® 647 (1:200) \| Life Technologies, Darmstadt, GER \| Catalogue #A-31573 RRID: AB_2536183 \| \| Goat anti-tdTomato (1:6000) \| Origene Europe, Herford, GER \| Catalogue #AB8181-200 RRID: AB_2722750 \| \| Guinea pig anti VGluT1 (1:1600) \| Synaptic Systems, Göttingen, GER \| Catalogue #135 304 RRID: AB_887878 \| \| Mouse anti S100B Antibody (SA-12) (1:2000) \| Novus, Abingdon, UK \| Catalogue #NBP1-41373 RRID: AB_2184561 \| \| Mouse anti-NeuN (1:500) \| Merck Millipore, Darmstadt, GER \| Catalogue #MAB377 RRID: AB_2298772 \| \| Rabbit anti EAAT2 (1:2000) \| Abcam, Cambridge, UK \| Catalogue #ab41621 RRID: AB_941782 \| \| Rabbit anti Iba1 (1:1000) \| Wako Chemicals GmbH, Neuss, GER \| Catalogue #019-19741 RRID: AB_839504 \| \| ***Plasmids*** \|  \|  \| \| pAAV-gfaABC1D-mRuby3 \| This study \|  \| \| pAAV-gfaABC1D-mRuby3 EAAT2 \| This study \|  \| \| pAAV-gfaABC1D-mRuby3 EAAT2 4KR \| This study \|  \| \| pAAV-gfaABC1D-mRuby3 EAAT2 S506X \| This study \|  \| \| pAAV-gfaABC1D-mYFP \| This study \|  \| \| pAAV-gfaABC1D-mYFP EAAT2 \| This study \|  \| \| pAAV-gfaABC1D-mYFP EAAT2 S506X \| This study \|  \| \| pCI-syn-iGluu \| Addgene, Watertown, MA, USA \| Catalogue #106122 \| \| pKanCMV-mRuby3-10aa-H2B \| Addgene, Watertown, MA, USA \| Catalogue #74258 \| \| pRcCMV mYFP EAAT2 (K320) \| Gifts from C. Fahlke and A.Baumann, Jülich, GER \|  \| \| pRcCMV mYFP EAAT2 S506X (K103) \| Gifts from C. Fahlke and A.Baumann, Jülich, GER \|  \| \| ***Primers for site-directed mutagenesis*** \|  \|  \| \| GLT1-K517R-Fw \| gatattgaaatgaccaGgactcaatccatttatg \| Modified from González-González et al. 2008 \| \| GLT1-K517R-Rw \| cataaatggattgagtcCtggtcatttcaatatc \| Modified from González-González et al. 2008 \| \| GLT1-K526R-Fw \| catttatgatgacatgaGgaaccacagggaaag \| Modified from González-González et al. 2008 \| \| GLT1-K526R-Rw \| ctttccctgtggttcCtcatgtcatcataaatg \| Modified from González-González et al. 2008 \| \| GLT1-K550R-Fw \| catagtagatgaatgcaGggtaactctggcag \| Modified from González-González et al. 2008 \| \| GLT1-K550R-Rw \| ctgccagagttaccCtgcattcatctactatg \| Modified from González-González et al. 2008 \| \| GLT1-K570R-Fw \| gaggaagaaccttggaGacgtgagaaataag \| Modified from González-González et al. 2008 \| \| GLT1-K570R-Rw \| cttatttctcacgtCtccaaggttcttcctc \| Modified from González-González et al. 2008 \| \| ***Recombinant virus strains*** \|  \|  \| \| pAAV PHP.eB-gfaABC1D-mRuby3 \| Charité - Viral Core Facility, Berlin, GER \|  \| \| pAAV PHP.eB-gfaABC1D-mRuby3 EAAT2 \| Charité - Viral Core Facility, Berlin, GER \|  \| \| pAAV PHP.eB-gfaABC1D-mRuby3 EAAT2 4KR \| Charité - Viral Core Facility, Berlin, GER \|  \| \| pAAV PHP.eB-gfaABC1D-mRuby3 EAAT2 S506X \| Charité - Viral Core Facility, Berlin, GER \|  \| \| pAAV PHP.eB-gfaABC1D-mYFP \| Charité - Viral Core Facility, Berlin, GER \|  \| \| pAAV PHP.eB-gfaABC1D-mYFP EAAT2 \| Charité - Viral Core Facility, Berlin, GER \|  \| \| AAV9-CamKII-iGluSnFRf-u #38698 \| UPenn Vector core, Pennsylvania, USA \|  \| \| ***Chemicals, Reagents*** \|  \|  \| \| 10x Roti-block solution \| Carl Roth GmbH, Karlsruhe, GER \| Catalogue #A151.1 \| \| APV \| Abcam, Cambridge, UK \| Catalogue #ab120271 \| \| Bicuculline methodide \| Sigma-Aldrich, Taufkirchen, GER \| Catalogue #14343-50MG \| \| Carbenoxolone \| Abcam, Cambridge, UK \| Catalogue #ab143590-5g \| \| DNQX \| Tocris, Bristol, UK \| Catalogue #2312/50 \| \| Endopeptidase LysC \| Wako, Neuss, GER \|  \| \| GFP-trap magnetic agarose beads \| ChromoTek, Planegg-Martinsried, GER \| Catalogue #gtma-20 \| \| Ketamine \| Sigma-Aldrich, Taufkirchen, GER \| Catalogue #K2753-1G \| \| MK801 \| Sigma-Aldrich, Taufkirchen, GER \| Catalogue #M107-50mg \| \| Novex 0.45 µm nitrocellulose membrane \| ThermoFischer Scientific, Dreieich, GER \| Catalogue #LC2001 \| \| Novex bis-tris gradient gel 4-12% \| Abcam, Cambridge, UK \| Catalogue #NP0321BOX \| \| Pluronic F-127 \| Sigma-Aldrich, Taufkirchen, GER \| Catalogue #P2443-250G \| \| Ready Tector Solution A \| Candor Bioscience G,bH, Wangen, GER \|  \| \| ReadyTector \| CANDOR Bioscience GmbH, Wangen, GER \| Catalogue #730 040 \| \| Roche cOmplete protease inhibitor cocktail \| Sigma-Aldrich, Taufkirchen, GER \| Catalogue #4693132001 \| \| SBFI-AM \| ThermoFischer Scientific, Dreieich, GER \| Catalogue #S-1264 \| \| Sequencing grade trypsin \| Promega, Mannheim, GER \| Catalogue #V511A \| \| Tetrodotoxin \| Abcam, Cambridge, UK \| Catalogue #ab120054 \| \| TFB-TBOA \| Tocris, Bristol, UK \| Catalogue #2532/10 \| \| Trypsin \| Promega, Mannheim, GER \|  \| \| WesternBright ECL \| Biozym Scientific , Hessisch Oldendorf, GER \| Catalogue #R-03031-D25 / R-03025-D25 \| \| Xylazine \| Sigma-Aldrich, Taufkirchen, GER \| Catalogue #110677 \| \| ***Equipment*** \|  \|  \| \| Andor Zyla4.2 plus \| Oxford Instruments, Oxford, UK \|  \| \| Axio Examiner A1 \| Carl Zeiss AG, Oberkochen, GER \|  \| \| Axioscope 2 FS plus \| Carl Zeiss AG, Oberkochen, GER \|  \| \| Cryo-grinder set \| OPS Diagnostics, Lebanon, NJ, USA \| Catalogue #CG 08-02 \| \| DynaMag-2 magnet \| ThermoFischer Scientific, Dreieich, GER \|  \| \| Emission filter XF3086 \| Omega Optical, Brattleboro, VT, USA \|  \| \| EPC-8 \| List, Darmstadt, GER \|  \| \| FW 1000 filter wheel \| Applied Scientific Instrumentation, Eugene, OR, USA \|  \| \| High Performance Liquid Chromatography System \| ThermoFischer Scientific, Dreieich, GER \|  \| \| ITC-16 \| HEKA Elektronik, Lambrecht, GER \|  \| \| KinematicaPolytron PT1300D homogenizer \| ThermoFischer Scientific, Dreieich, GER \| Catalogue #10764233 \| \| Polychrome V \| Till Photonics, Planegg-Martinsried, GER \|  \| \| Proxeon nano-LC system \| ThermoFischer Scientific, Dreieich, GER \|  \| \| Reprosil 75 μm x 250 mm, 3 μm \| Dr. Maisch GmbH, Ammerbuch, GER \|  \| \| Spectrometer  Thermo Orbitrap Fusion Mass \| ThermoFischer Scientific, Dreieich, GER \|  \| \| UVICO ultraviolett or visible light source \| Rapp OptoElectronic, Hamburg, GER \|  \| \| ***Software*** \|  \|  \| \| ApE (A Plasmid Editor) \| M. Wayne Davis, Utah, UT, USA \| Version #10.55 \| \| ImagePro Plus \| MediaCybernetics, Roper, Sarasota, FL, USA \| Version #6.0 \| \| MaxQuant.Live \| MaxQuant \| Version #1.6.0.1 \| \| Perseus \| omicX \| Version# 1.6.2.1 \| \| Prism \| GraphPad, San Diego, CA, USA \| Version# 8 \| \| Solis \| Acal GmbH, Gröbenzell, GER \| Version# 4.30.30034.0 \| \| TIDA5.25 \| HEKA Elektronik, Lambrecht, GER \| Version# 5.25 \| \| UGA-42 Firefly \| Rapp OptoElectronic, Hamburg, GER \| N/A \| |  |  |  |  |  |  |  |  |  |  |  |
| --- | --- | --- | --- | --- | --- | --- | --- | --- | --- | --- | --- | --- | --- | --- | --- | --- | --- | --- | --- | --- | --- | --- | --- | --- | --- | --- | --- | --- | --- | --- | --- | --- | --- | --- | --- | --- | --- | --- | --- | --- | --- | --- | --- | --- | --- | --- | --- | --- | --- | --- | --- | --- | --- | --- | --- | --- | --- | --- | --- | --- | --- | --- | --- | --- | --- | --- | --- | --- | --- | --- | --- | --- | --- | --- | --- | --- | --- | --- | --- | --- | --- | --- | --- | --- | --- | --- | --- | --- | --- | --- | --- | --- | --- | --- | --- | --- | --- | --- | --- | --- | --- | --- | --- | --- | --- | --- | --- | --- | --- | --- | --- | --- | --- | --- | --- | --- | --- | --- | --- | --- | --- | --- | --- | --- | --- | --- | --- | --- | --- | --- | --- | --- | --- | --- | --- | --- | --- | --- | --- | --- | --- | --- | --- | --- | --- | --- | --- | --- | --- | --- | --- | --- | --- | --- | --- | --- | --- | --- | --- | --- | --- | --- | --- | --- | --- | --- | --- | --- | --- | --- | --- | --- | --- | --- | --- | --- | --- | --- | --- | --- | --- | --- | --- | --- | --- | --- | --- | --- | --- | --- | --- | --- | --- | --- | --- | --- | --- | --- | --- | --- | --- | --- | --- | --- | --- | --- | --- | --- | --- | --- | --- | --- | --- | --- | --- | --- | --- | --- | --- | --- | --- | --- | --- | --- | --- | --- | --- | --- | --- | --- | --- | --- | --- | --- | --- | --- | --- | --- | --- | --- | --- | --- | --- | --- | --- | --- | --- | --- | --- | --- | --- | --- | --- | --- | --- | --- | --- | --- | --- | --- | --- | --- | --- | --- | --- | --- | --- | --- | --- | --- | --- | --- | --- | --- | --- | --- | --- | --- | --- | --- | --- | --- | --- | --- | --- | --- | --- | --- | --- | --- |

**Supplemental Table 2**. **Definitions of Open Field Test variables.**

***Mouse centroid position***

It reflects the body positions in the horizontal plane and is defined as

$$X_{c}=\frac{\sum_{i=1}^{N} X_{i}\times{Pixel intensity}_{i}}{\sum_{i=1}^{N} {Pixel intensity}_{i}};Y_{c}=\frac{\sum_{i=1}^{N} Y_{i}\times{Pixel intensity}_{i}}{\sum_{i=1}^{N} {Pixel intensity}_{i}}$$

*Xc, Yc is the position centroid. N - the number of suprathreshold pixels, i – pixel index. Xi, Yi – pixel coordinates with corresponding pixel intensity.*

***Virtual mouse radius (v.m.r.)***

*Virtual mouse radius*$= \sqrt{\frac{1}{\pi}\times N_{suprathreshold pixels}\times Pixel area}$

***Start point***

*Start point = С(X_c_,Y_c_) |√((X_Stop_-X_c_ )^2^+(Y_Stop_-Y_c_ )^2^ )>1×Virtual mouse radius*

*where C(Xc, Yc) - is the current centroid position and Xstop, Ystop – the coordinates of the preceding stop point.*

***Stop point***

*Stop point = C(X_c_,Y_c_)|(√((X_c_-X_break_^2^+(Y_c_-Y_break_^2^ )<1×Virtual mouse radius)U(С(X_c_,Y_c_)=С(X_0_,Y_0_))*

*where C(X_0_, Y_0_) is the centroid position of the first frame and X_break_, Y_break_ – the coordinates of the mouse centroid at the moment when it lags the current position by the time of running start.*

***The running start episode***

is an initial component of the open field locomotion between a start and a stop point.

*Running start episode = {C(X_c_,Y_c_)ϵRunning episode|√((X_c_-X_Stop_)^2^+(Y_c_-Y_Stop_ )^2^)>1×Virtual mouse radius∩ √((X_c_-X_Stop_ )^2^+(Y_c_- Y_Stop_)^2^ )<2×Virtual mouse radius}*

*where Xstop, Ystop are the coordinates of the preceding stop point.*

***The running episode***

is a component of the open field locomotion between a start and a stop point.

*Running episode = {C(X_c_,Y_c_)ϵTrajectory[Start point, next Stop point)}*

***The resting episode***

is a component of open field locomotion between a stop and a start point.

*Resting episode = {C(X_c_,Y_c_) ϵTrajectory[Stop point, Start point)}*

***Mean running velocity***

$$Mean running velocity=\frac{\sum_{i=1}^{T} {Distance travelled during the running episode}_{i}}{\sum_{i=1}^{T} {Duration of running episode}_{i}}$$

*where T - the number of running episodes duringthe 5 min observation period; i - index of the running episode.*

***Mean resting velocity***

$$Resting velocity=\frac{\sum_{i=1}^{R} {Position change during the resting episode}_{i}}{\sum_{i=1}^{R} {Duration of resting episode}_{i}}$$

*where R - the number of resting episodes during the 5 min observation period; i - index of the resting episode.*

***The resting area***

is a circular area centered to the origin of a radius equaling the maximal deviation from the center point.

***The center point of the resting area***

$$X_{center}=\frac{\sum_{i=1}^{N} X_{i}}{N};Y_{center}=\frac{\sum_{i=1}^{N} Y_{i}}{N}$$

*where Xcenter and Ycenter are the coordinates of the center point; N – the number of centroid positions contributing to the resting episode; i – index of centroid value at rest; Xi and Yi - actual coordinates during the resting episode.*

***Maximal deviation from the resting area center point***

$$Maximal deviation from center point=Max\left( \sqrt{\left( X_{i}-X_{center} \right)^{2}+\left( Y_{i}-Y_{center} \right)^{2}} \right)$$

***Mean deviation from the resting area center point***

$$Mean deviation from center point=Mean\left( \sqrt{\left( X_{i}-X_{center} \right)^{2}+\left( Y_{i}-Y_{center} \right)^{2}} \right)$$

***Open field resting time***

%Fraction of the observation time occupied by resting episodes

***Open field center time***

%Fraction of the observation time spent in the central area of the open field

**SUPPLEMENTAL TEXT AND FIGURES**

**Assessment of spontaneous locomotor activity in WT and HD mice**

The open field test (OFT) is a frequently used but not highly standardized tool to evaluate spontaneous locomotor activity (Wahlsten, 2001;Kostrzewa and Kas, 2014). Originally it was developed for the characterization of behavioral phenotyping of rodents exhibiting symptoms of anxiety. Later on, it was used for the quantification of symptoms and recovery in rodents with experimental lesions, intoxication and neurodegenerative disease (Crawley, 2003), taking advantage of automated analysis of positional data from movement trackers (Tatem et al., 2014). In many cases the OFT provided not much more than the distance travelled during the time of observation, but a relatively recent study in CAG140 knock-in mice extracted 6 indicators characterizing the locomotion in HET of 52 weeks and older (Fowler and Muma, 2015). These indicators were: distance travelled (1), force variability (2), number of wall rears (3), wall rear duration (4), number of low mobility bouts (5) and number of long strait runs (6). Significant deficits were found in 1, 3, 5, 6. The algorithm used to determine the number of "mobility bouts" and number of "long strait runs" is perhaps similar to our measure "incidence of starts" (as the number of starts in 5 min). We also determined the respective movement velocities and the actual extension of the "bouts". The results were correlated with a new test, the step-over latency test (SOLT), a test to some extent corresponding to the number of wall rears (3) in the Fowler and Muma study. Supplemental Tab. 1 presents the definitions of the analyzed OFT variables. More details are described in the Methods section.

The SOLT is associated with, but not identical to, the OFN. It quantifies the time needed to climb the walls of a large Petri dish (see Methods). In principle, it might be understood as the time needed to initiate exploratory behavior from a forced position in the center of a small platform. However, more work is needed to clarify possible contributions of other determinants of spontaneous motor behavior, first of all anxiety. While HD mice spend significantly less time in the center of an open field, they may need more time to escape from such position to the more comfortable peripheral parts of the open field.

As all behavioral data presented in the main text are derived from mice with viral injections into the brain it seemed useful to compare some of the obtained results with data from noninjected mice. Supplemental Fig.1 presents the outcome of SOLT in noninjected WT and HET at an average age of 59 weeks (HET), i.e. about the same age as the injected mice (compare Fig. 2). No significant differences were found in the step-over latencies of CTRL-injected and non-injected WT and HET.


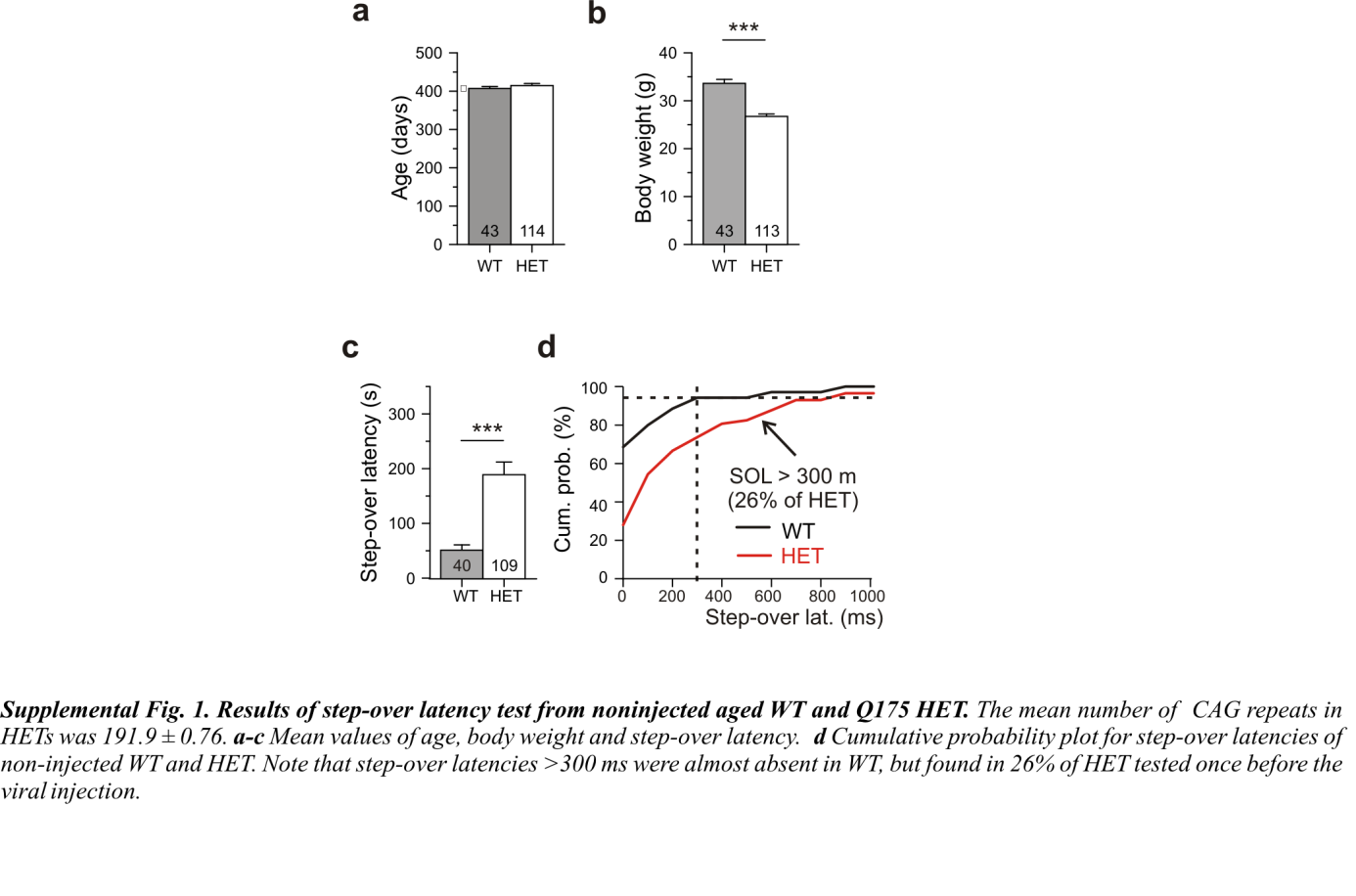


**Partial recovery of astrocytic glutamate uptake after systemic application of the EAAT2-S506X vector, but no improvement of locomotion**

By chosing intrastriatal injections of the EAAT2 vectors one naturally neglects astrocyte pathologies in other brain areas, including those of the subthalamic nucleus (Atherton et al., 2016), a target of the hyperdirect pathway. Quite likely, one can expect additional or even different effects after systemic application of a given vector. An additional series of experiments was therefore carried out to test the effect of systemic vector application. The animal groups were the same as in Fig. 6, 8 (intrastriatal injections) but now EAAT2-S506X and the respective CTRL vectors were injected via the tail vein.

The SBFI recordings were only performed in mRuby+ astrocytes. The transgene-induced fluorescence was much lower than in mice with direct injection in the striatum. The brightest astrocytes were chosen for an estimation of glutamate uptake. The results (Supplemental Fig. 2a, b) suggest that in the selected astrocytes glutamate uptake activity was again higher in the HET:EAAT2-S506X group compared to HET:CTRL. But none of the quantified locomotor indicators showed a significant effect of HET-S506X group (Supplemental Fig. 2c-g). Moreover, the WT:CTRL values obtained in the OFT test (both total distance travelled and center time) were significantly lower than in WT:CTRL mice injected in the striatum. These results hint the possibility that the systemic vector application might have some side effects.


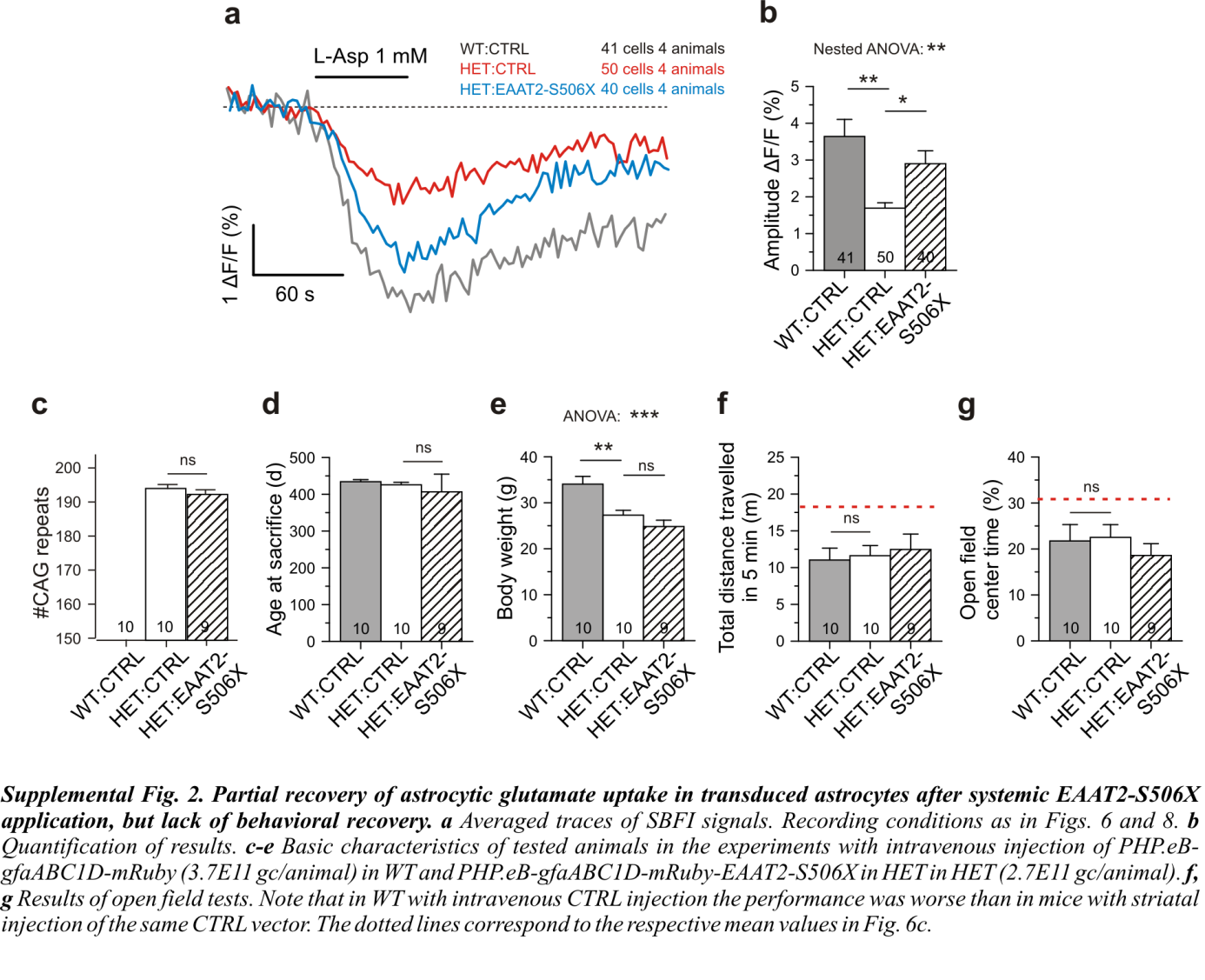


**Rescue of neuron excitability and astroglial GABA release**

It is already well known that HD progression is associated with an increase in the excitability of striatal projection neurons of the dorsal striatum (SPNs), and it was shown that nonsynaptic release of GABA from astrocytes can contribute to the underlying pathogenic mechanism. The enhanced excitability of striatal neurons is reflected in the appearance of pathological activity patterns (Rothe et al., 2015), lower firing thresholds of individual SPNs and higher values of their whole-cell input resistance R_N_ (Dvorzhak et al., 2013). Previous studies from our lab suggested that nonsynaptic release of GABA from mHTT-expressing astrocytes could contribute to these changes (Wojtowicz et al., 2013).

A similar approach was now used to record the membrane currents of SPNs surrounded by YFP-tagged astrocytes to determine whether changes in R_N_ could contribute to the phenotype of aged Q175 HET. This was the case (Supplemental Fig. 3a, b). Moreover, EAAT2-S506X-treated HET exhibited a tendency to change towards WT levels, although a larger number of tests were needed to confirm this possibility with a respective posthoc test.

Patch clamp recordings were further performed to record the tonic GABA(A) receptor currents of SPNs, I_Tonic(GABA)_ (Supplemental Fig. 3c). This current reflects the ambient GABA concentration and, in part, depends on spontaneous release of GABA from astrocytes. The level of extrasynaptic GABA-dependent chloride conductance is believed to play a major role in the control of neuron excitability (Kersante et al., 2013). Due to the co-localization of EAAT2 and GAT3 at the sites of glutamate release and the fact that glutamate transport via EAAT2 can influence the driving forces for GAT3 (Heja et al., 2019;Dvorzhak et al., 2013), a weakness of EAAT2 may lead to a reduction of astrocytic GABA release via GAT3 and, hence, smaller tonic inhibition of SPNs (Wojtowicz et al., 2013). The present results (Supplemental Fig. 3d) are consistent with this hypothesis although the difference between CTRL- and EAAT2-S506X-treated HET failed to reach the level of statistical significance with nested ANOVA.


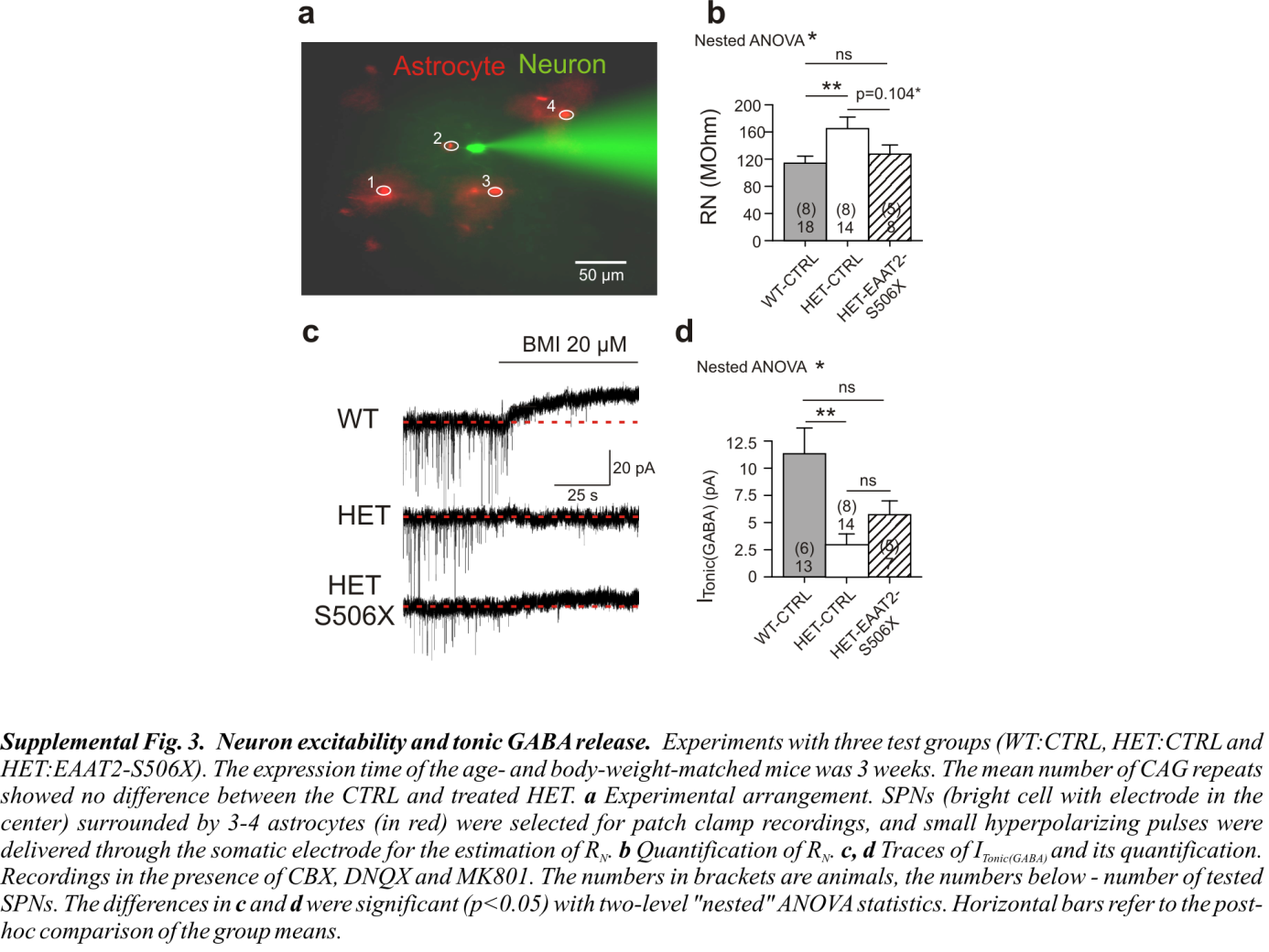


**REFERENCE LIST**

Atherton JF, McIver EL, Mullen MR, Wokosin DL, Surmeier DJ, Bevan MD (2016) Early dysfunction and progressive degeneration of the subthalamic nucleus in mouse models of Huntington's disease. Elife 5: 10.

Crawley JN (2003) Behavioral phenotyping of rodents. Comp Med 53: 140-146.

Dvorzhak A, Semtner M, Faber DS, Grantyn R (2013) Tonic mGluR5/CB1-dependent suppression of inhibition as a pathophysiological hallmark in the striatum of mice carrying a mutant form of huntingtin. J Physiol 591: 1145-1166.

Fowler SC, Muma NA (2015) Use of a force-sensing automated open field apparatus in a longitudinal study of multiple behavioral deficits in CAG140 Huntington's disease model mice. Behav Brain Res 294: 7-16.

Heja L, Simon A, Szabo Z, Kardos J (2019) Feedback adaptation of synaptic excitability via Glu:Na(+) symport driven astrocytic GABA and Gln release. Neuropharmacology 10.

Kersante F, Rowley SC, Pavlov I, Gutierrez-Mecinas M, Semyanov A, Reul JM, Walker MC, Linthorst AC (2013) A functional role for both -aminobutyric acid (GABA) transporter-1 and GABA transporter-3 in the modulation of extracellular GABA and GABAergic tonic conductances in the rat hippocampus. J Physiol 591: 2429-2441.

Kostrzewa E, Kas MJ (2014) The use of mouse models to unravel genetic architecture of physical activity: a review. Genes Brain Behav 13: 87-103.

Rothe T, Deliano M, Wojtowicz AM, Dvorzhak A, Harnack D, Paul S, Vagner T, Melnick I, Stark H, Grantyn R (2015) Pathological gamma oscillations, impaired dopamine release, synapse loss and reduced dynamic range of unitary glutamatergic synaptic transmission in the striatum of hypokinetic Q175 Huntington mice. Neurosci 311: 519-538.

Tatem KS, Quinn JL, Phadke A, Yu Q, Gordish-Dressman H, Nagaraju K (2014) Behavioral and locomotor measurements using an open field activity monitoring system for skeletal muscle diseases. J Vis Exp 51785.

Wahlsten D (2001) Standardizing tests of mouse behavior: reasons, recommendations, and reality. Physiol Behav 73: 695-704.

Wojtowicz AM, Dvorzhak A, Semtner M, Grantyn R (2013) Reduced tonic inhibition in striatal output neurons from Huntington mice due to loss of astrocytic GABA release through GAT-3. Front Neural Circuits 7: 188-199.
